# Heterogeneity in the entire genome for three genotypes of peach [*Prunus persica* (L.) Batsch] as distinguished from sequence analysis of genomic variants

**DOI:** 10.1186/1471-2164-14-750

**Published:** 2013-11-01

**Authors:** Jonathan Fresnedo-Ramírez, Pedro J Martínez-García, Dan E Parfitt, Carlos H Crisosto, Thomas M Gradziel

**Affiliations:** Department of Plant Sciences, University of California Davis, One Shields Ave, Davis, CA 95616 USA

## Abstract

**Background:**

Peach [*Prunus persica* (L.) Batsch] is an economically important fruit crop that has become a genetic-genomic model for all *Prunus* species in the family Rosaceae. A doubled haploid reference genome sequence length of 227.3 Mb, a narrow genetic base contrasted by a wide phenotypic variability, the generation of cultivars through hybridization with subsequent clonal propagation, and the current accessibility of many founder genotypes, as well as the pedigree of modern commercial cultivars make peach a model for the study of inter-cultivar genomic heterogeneity and its shaping by artificial selection.

**Results:**

The quantitative genomic differences among the three genotypes studied as genomic variants, included small variants (SNPs and InDels) and structural variants (SV) (duplications, inversions and translocations). The heirloom cultivar 'Georgia Belle’ and an almond by peach introgression breeding line 'F8,1-42’ are more heterogeneous than is the modern cultivar 'Dr. Davis’ when compared to the peach reference genome ('Lovell’). A pair-wise comparison of consensus genome sequences with 'Lovell’ showed that 'F8,1-42’ and 'Georgia Belle’ were more divergent than were 'Dr. Davis’ and 'Lovell’.

**Conclusions:**

A novel application of emerging bioinformatics tools to the analysis of ongoing genome sequencing project outputs has led to the identification of a range of genomic variants. Results can be used to delineate the genomic and phenotypic differences among peach genotypes. For crops such as fruit trees, the availability of old cultivars, breeding selections and their pedigrees, make them suitable models for the study of genome shaping by artificial selection. The findings from the study of such genomic variants can then elucidate the control of pomological traits and the characterization of metabolic pathways, thus facilitating the development of protocols for the improvement of *Prunus* crops.

**Electronic supplementary material:**

The online version of this article (doi: 10.1186/1471-2164-14-750) contains supplementary material, which is available to authorized users.

## Background

High-throughput DNA sequencing has made available large quantities of genomic information allowing a more complete characterization of genomes at the chromosome level. This approach, which has been successfully applied to human genomics through The 1000 Genomes Project Consortium project [1], shows similar promise for the genetic analysis and improvement of crop species [2].

Comparative genomics has been used to distinguish intraspecific differences such as among different agronomic cultivars. Recently, determination of the genome sequences of important tree crops promises to advance genomic analysis of these perennial and clonally propagated crops to the genomic analysis levels now routine for agronomic crops such as rice (*Oryza sativa* L.) and maize (*Zea mays* L.).

Unlike sexual seed propagation common to agronomic crops, most fruit tree crops, such as *Prunus* species, are propagated through vegetative methods; this permits the capture of the individual genetic and epigenetic composition, including chromosomal variants, which may play important roles in their genetic improvements and even domestication.

Peach [*Prunus persica* (L.) Batsch] has become a model species for genetic and genomic studies in the Rosaceae because it has several characteristics facilitating genetic studies, including: important genes described and mapped, a small diploid genome [3], self-compatibility, and a short juvenile period. As a result of the International Peach Genome Initiative (IPGI), a peach reference genome sequence has been obtained [4]. The peach genome size is approximately 227.3 million base pairs (227.3 MB), and its eight main scaffolds align with the eight linkage groups in the reference physical genetic map developed for peach, which was generated from an F_2_ progeny of an interspecific cross between peach and almond [5–8]. The publically available peach genome sequence shows high correspondence to the previous physical map obtained for peach [9, 10]. The reference genome is based on a doubled-haploid sample of the 'Lovell’ cultivar [9], which was chosen as the preferred model for pursuing several types of genetic and genomic studies since all of the alleles are represented as homozygous. Peach possesses a haploid chromosome set of eight chromosomes [11]. The eight principal scaffolds of the genome sequence are concordant with the eight linkage groups of the peach physical and genetic maps. 'Lovell’ exhibits the typical phenotype of domesticated peach, which has yellow flesh, yellow skin with around 15% blush, detached pit (freestone), and a melting type flesh texture, with some red pigmentation around the pit (Zhebentyayeva, manuscript in preparation).

Peach, a species domesticated over 4000 years ago [12], exhibits high phenotypic variability but restricted genetic diversity. Low genetic diversity is a consequence of the self-compatibility in peach [13], as well as a recent genetic-bottleneck during the development of modern European and American cultivars [14].

Chromosome 1 is the largest and sub-metacentric, chromosomes 2 and 4 to 7 are metacentric, while chromosomes 3 and 8 are acrocentric. Chromosome 8 is the shortest. Chromosomes 6 and 7 are nucleolus-organizers [15, 16]. Techniques such as fluorescence *in situ* hybridization (FISH) in almond, which has high chromosomal synteny with peach [17], has led to the identification of each chromosome based on the positions of ribosomal DNA genes [18, 19]. Most current cultivars have been developed in the last 100 to 150 years [20]. Because of the low genetic diversity among cultivars [13], the sequence of an individual genome should be representative of the general genic organization in peach.

While several protocols for genetic transformation had been reported for this species [14, 21–23]; an efficient standardized transformation system is not yet available for the species [24]. The consequent limitation on detailed genome annotation further emphasizes the value of genome sequencing as a promising approach for genomic analysis and manipulation.

The genome sequences of three different genotypes of peach were sequenced at the University of California, Davis [25] and aligned to the 'Lovell’ peach reference genome. 'Lovell’ is a double haploid line developed with colchicine by Toyama [26]. The accessions consisted of the heirloom fresh-market cultivar 'Georgia Belle’ (also known as 'Belle of Georgia’), the modern processing cultivar 'Dr. Davis’ and the almond breeding introgression line 'F8,1-42’ from the Processing Peach Breeding Program at UC Davis. These accessions were selected because of their commercial relevance, historic context, diverse phenotypes, and the generation of mapped progenies from these parent cultivars.

The discovery and quantification of genomic variants enables researchers to characterize genomic differences among specific genotypes. For clonally propagated crops, such as peach, individual genotypes or clones can represent a large proportion of the commercial acreage around the world. Genomic variants include both changes in the nucleotides as well as changes in chromosome structure. For trait mapping, nucleotide variants, such as Single Nucleotide Polymorphisms (SNPs, in which one nucleotide is substituted for another) are commonly studied. Insertions and Deletions (InDels, i.e. the addition or loss of a number of nucleotides in a chain no longer than 50) are commonly used to study evolutionary divergence and speciation. Genomic rearrangements (or chromosomal rearrangements) longer than 50 nucleotides are often considered structural variants (SV) [27] since they have a direct impact on the structure and behavior of the chromosomes as well as causing variations in gene dosage. Such structural variants are the result of rearrangements within a chromosome or between chromosomes. While the importance of such variation is recognized in plants, their study remains limited. Typical sources of variation include insertions (longer than 50 bp), inversions, duplications, translocations, and, where they have been characterized, mobile-elements in the target genome, or a combination of such events in balanced or unbalanced signatures [27].

Analysis of SNPs and InDels has become common in genetic and genomic studies such as genetic linkage maps and Quantitative Trait Loci (QTL). In addition to their frequency, they provide information concerning recombination, selection, divergence and genetic structure. In human studies, structural variants have increasingly been considered as a major driving force in evolution [28]. Structural variations are the main source of genomic variation, having been associated with important phenotypic changes, including several rare and complex diseases in humans [27]. The association between structural variants and associated phenotypes in plants has been less thoroughly studied, except for maize [29] with comparisons among inbreed lines [30] and a comparison with teosinte (*Zea mays* ssp. *parviglumis* H.H.Iltis & Doebley) [31]. Recent studies have shown this variation to be associated with changes of Copy Number Variation (CNV) in Arabidopsis [32] and intra-cultivar variation in soybean [*Glycine max* (L.) Merr.] [33, 34]. The discovery and quantification of genomic variants can be used in comparative genomics in order to estimate the genomic heterogeneity among genotypes of the same species, including different cultivars and even different clones of the same cultivar.

Methods of phylogenetic reconstruction which take advantages of powerful statistical approaches and mathematical models, have become indispensable tools in describing the patterns of DNA base substitution, amino acid replacement, and the structural differences among genomes [35]. The use of methods such as the genome conservation matrix [36] enables researchers to make quantitative measurements of comparison among and between genomes, and the application of these measurements to the study of inter-cultivar genome differences is particularly valuable.

The ready availability of genomic and genetic information generated by high-throughput sequencing allows the application of advanced bioinformatic methods to characterize the quantity and distribution of the small and structural variants, and so clarify the effects of such genomic variants.

Genome heterogeneity among three peach genotypes was studied through the discovery and quantification of genomic variants, including small variants, such as SNPs and InDels, and structural variants, such as inversions, duplications and translocations, to better understand the quantitative differences in the genome sequences and their relationship to the number, type and impact of variants. The implications for improved understanding of peach genomics and genetic improvement are discussed. Because desirable genetic and epigenetic genomic variation can be captured in clonally propagated crops such as peach, unique opportunities for clonal crop improvement are possible.

## Results

### Small variants

**Figure 1:**
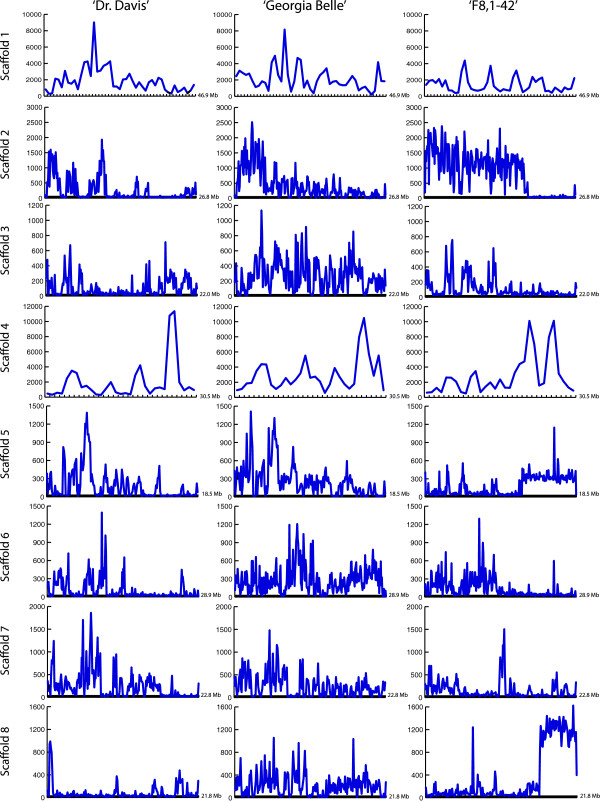
**Comparison in the frequency distribution of the variants along each scaffold for 'Dr. Davis’, 'Georgia Belle’, and 'F8,1-42’.** The frequency is given in number of variants per 100 Kb for a particular position in the scaffold.

Most common small variants (SNPs, InDels) for the three genotypes are summarized in Table 1 and compared with the genome reference sequence. The most common variants were SNPs. Insertions and Deletions were present in similar numbers among the three genotypes, and proportionally, these variants represent approximately 8% of the small variants in 'F8,1-42’, 9% in 'Georgia Belle’ and 10% in 'Dr. Davis’. The distribution and frequency of the variants among the eight scaffolds is shown in Figure 1. The differences in small variants exhibited among the genotypes and among the chromosomes were evident, the most distinct being the high frequency of variants in 'F8,1-42’ at the end of chromosomes 4 and 8, and the particular pattern of variation exhibited at the end of chromosome 5, suggesting possible chromosomal rearrangements in this genotype.

**Table 1:** **Total number of variants, type and zygosity of variants for each genotype**

Genotype	Total	SNPs		Insertions		Deletions	
		***Hom***	***Het***	***Hom***	***Het***	***Hom***	***Het***
**'Georgia Belle’**	639,062	581,616		27,515		29,931	
		*2,910*	*578,706*	*7,745*	*19,770*	*7,790*	*22,141*
**'Dr. Davis’**	399,649	358,648		19,148		21,853	
		*1,428*	*357,220*	*6,756*	*12,392*	*6,995*	*14,858*
**'F8,1-42’**	593,720	546,542		22,543		24,635	
		*3,698*	*542,844*	*8,617*	*13,926*	*8,674*	*16,159*

“Hom” refers to homozygous variants and “Het” to heterozygous variants.

The heirloom cultivar 'Georgia Belle’ exhibited the greatest variation with respect to the 'Lovell’ reference genome, followed by the breeding introgression line 'F8,1-42’ and then the modern cultivar 'Dr. Davis’. A similar pattern was followed for each type of small variants, as well as for zygosity. The genome-wide change rate for 'Georgia Belle’ was 1 change for every 355 bases, 1 for every 382 for 'F8-1.42’ and 1 for every 568 for 'Dr. Davis’.

The output of SnpEff 3.0c (see Additional files 1, 2 and 3) provided detailed information on the number of changes and the change rate per chromosome (scaffolds as denominated by the Peach Genome Initiative). Among the eight scaffolds that comprise the genome of peach, the highest change rate was observed in scaffold 2. This finding was observed for all three genotypes, with one change for every 122 bases for 'F8,1-42’, one change for every 235 bases for 'Georgia Belle’, and one change for every 397 bases for 'Dr. Davis’. Interestingly, scaffold 8 in 'Dr. Davis’ shows the lowest rate of change, with one change for every 1268 bases, followed by scaffold 5 of 'F8,1-42’, which exhibits one change for every 1111 bases. Also, notable is that the change rate for the eight scaffolds of 'Georgia Belle’ ranges from 235 to 462, while for 'F8,1-42’, it is between 122 and 1111 and for 'Dr. Davis’ it is 392 and 1268.

'Georgia Belle’ exhibits the highest proportion of heterozygous versus homozygous variants (97.1%), followed by 'F8,1-42’ (96.5%) and then 'Dr. Davis’ (96.2%). SnpEff also evaluated the impact of the changes based on the known annotation for the peach reference genome. Around 95% of the changes reported by genotype were considered sequence modifiers; the remaining ~5% consisted of moderate impact (~2.68% avg.), low impact (~1.85% avg.) and high impact (~0.28%) changes in the transcript unit. Few high impact variants were reported for each genotype, being greater for 'F8,1-42’ and 'Georgia Belle’, both with over 2000 changes. A total of 2729 changes were considered high impact changes in 'F8,1-42’ (0.281% of the total number of changes), 2277 in 'Georgia Belle’ (0.221%), and 1691 (0.268%) in 'Dr. Davis’.

For the three effects per functional class (missense, nonsense and silent), the three genotypes showed between 57 and 59% missense changes, 38.85 and 40.3% silent changes, and a very small proportion of nonsense changes, ranging between 1.403 and 1.88%. The Missense/Silent ratio for 'Dr. Davis’ is 1.5262, 1.4481 for 'Georgia Belle’ and 1.4347 for 'F8,1-42’.

SnpEff also provided a detailed summary of the occurrence of small variants by type (Table 2) and by genomic region (Table 3, the two tables are complementary). The most common type of change is Non-Synonymous-Coding change, which ranges in each genotype between 2.5 and 3% of the total changes. Synonymous Coding changes were the next most common type of change, ranging between 1.6 and 2%. The remaining types of changes were present in low frequencies, since these do not exceed 0.14%. Changes such as Frame Shift surpass 1000 events in 'Georgia Belle’ (1,134) and in 'F8,1-42’ (1,284), while the lowest frequency change was the Non-Synonymous-Start type, with less than 10 events per genotype.

**Table 2:** **Count and percentage of changes given by small variants by type of change for each genotype**

Type of change (alphabetical order)		'Georgia Belle’		'Dr. Davis’		'F8,1-42’
	Count	Percent	Count	Percent	Count	Percent
**Codon Change + Codon Deletion**	98	0.01%	64	0.01%	95	0.01%
**Codon Change + Codon Insertion**	125	0.012%	79	0.013%	131	0.013%
**Codon Deletion**	143	0.014%	82	0.013%	135	0.014%
**Codon Insertion**	56	0.005%	35	0.006%	63	0.006%
**Frame Shift**	1,134	0.11%	847	0.134%	1,284	0.132%
**Non-Synonymous-Coding**	25,607	2.489%	15,537	2.464%	28,699	2.953%
**Non-Synonymous-Start**	6	0.001%	2	0.0005%	4	0.0001%
**Start Gained**	258	0.025%	169	0.027%	211	0.032%
**Start Lost**	49	0.005%	35	0.006%	42	0.004%
**Stop Gained**	635	0.062%	499	0.079%	947	0.097%
**Stop Lost**	75	0.007%	45	0.007%	70	0.007%
**Synonymous Coding**	17,743	1.725%	10,217	1.62%	20,046	2.062%
**Synonymous Stop**	25	0.002%	16	0.003%	38	0.004%

**Table 3:** **Count and number of changes per genomic region in each genotype**

Region (alphabetical order)		'Georgia Belle’		'Dr. Davis’		'F8,1-42’
	Count	Percent	Count	Percent	Count	Percent
**Downstream**	351,984	34.216%	210,781	33.431%	332,654	34.226%
**Exon**	45,696	4.442%	27,458	4.355%	51,554	5.304%
**Intergenic**	162,860	15.831%	108,303	17.178%	147,753	15.202%
**Intron**	79,677	7.745%	47,897	7.597%	82,602	8.499%
**Splice site acceptor**	191	0.019%	121	0.019%	183	0.019%
**Splice site donor**	193	0.019%	144	0.023%	203	0.021%
**Upstream**	382,086	37.142%	231,850	36.773%	349,884	35.998%
**UTR-3′**	3,863	0.376%	2,430	0.385%	4,602	0.473%
**UTR-5′**	2,168	0.211%	1,504	0.239%	2,507	0.258%

Most changes were downstream (33-34%) and upstream (36-37%) of the genes included in the annotation of the peach genome reference. The changes in the intergenic regions of the genomes account for 15-17% of the total, while the changes in introns represented between 7.6 and 8.5% of the changes. The portion of changes within the exonic regions ranged between 4.35 and 5.30%; 'F8,1-42’ showed 51,554 changes (5.304%), while 'Georgia Belle’ showed 45,696 (4.442%) and 'Dr. Davis’ 27,458 (4.355%). Changes occurring within the Untranslated Regions (UTR) 3′ and 5′ were present in proportions between 0.211 and 0.473%.

The base change from guanine (G) to adenine (A) was the most common in 'Georgia Belle’ and 'Dr. Davis’, with 96,058 and 59,129 changes, respectively. Most changes were from cytosine (C) to thymine (T) in 'F8,1-42’. In all cases, changes were denominated as transitions. The total number of transitions and transversions per genotype, as well as their respective Transitions/Transvertion (Ti/Tv) ratios, were presented in Table 4. All three genotypes exhibited Ti/Tv ratios above 3, with 'Georgia Belle’ showing a value above 3.6.

**Table 4:** **Number of transitions and transversions per genotype**

	'Georgia Belle’	'Dr. Davis’	'F8,1-42’
**Transitions**	374,886	227,722	339,879
**Transversions**	206,730	130,926	206,663
**Ti/Tv ratio**	3.6268	3.4786	3.2892

Ti/Tv is a ratio of rates, not of observed events. Since transitions are two times more frequent than transversions, the Ti/Tv ratio is twice the ratio of events = 2×(Ti/Tv).

For codon changes (based in SNPs), 'F8,1-42’ exhibited CCG (Proline) to CCA (Proline) as the most common change (325 events), which results in a synonymous change in transcription. The most common non-synonymous codon change was that from GAG (Glutamic Acid) to AAG (Lysine), with 309 events. 'Georgia Belle’ exhibited AAG (Lysine) to AAA (Lysine) as the most common synonymous codon change (306 events), and GGA (Glycine) to AAA (Lysine) as the most common non-synonymous change with 282 events. 'Dr. Davis’ exhibited GGA (Glycine) to AAA (Lysine) as the most common non-synonymous codon change, with 183 events, and AAC (Asparagine) to AAT (Asparagine) as the most common synonymous codon change with 176 events.

The most common amino acid changes per genotype were: Alanine to Valine, 666 times in 'F8,1-42’, followed by 655 Valine to Isoleucine events, and 603 Alanine to Tyrosine events. For 'Georgia Belle’, the change from Alanine to Valine occurs 553 times, followed by the change from Valine to Isoleucine, with 523 events, and 497 changes from Alanine to Tyrosine. Finally, 'Dr. Davis’ exhibits 352 changes from Glutamic acid to Lysine, followed by Alanine to Tyrosine, with 351 changes, and 349 Alanine to Valine changes.

### Structural variants

Two hundred and ninety two significant structural variants were identified from the comparisons of the three peach genotypes with the 'Lovell’ reference genome. The longest structural variant was a balanced inversion of a genomic fragment (Bal-Inv-Framt) in 'Georgia Belle’ at 1075 bp (variant ID 69,825 in Table 5).

**Table 5:** **Exclusive Structural Variants per genotype, their length, their type and the genomic region in which they occurred**

'Dr. Davis’						
**ID**	**Scaffold**	**Coordinates**	**SV Type**	**Length**	**Sequence**	**Gene or Repeat**
1495	1	13799591..13800210	UnBal-Inv-Dup	619	Gene	ppb020139m.g
16911	2	10443723..10444191	UnBal-Inv-Dup	468	-	-
17043	2	10707357..10707900	UnBal-Inv-Dup	543	Repeat	Repeat_45491
19815	2	17082238..17082630	Bal-Inv-Trans	392	Repeat	Repeat_50409, Repeat_50410, Repeat_50411
19815	3	5906870..5907047	Bal-Inv-Trans	177	Repeat	Repeat_61206
20201	2	1815521..1816145	UnBal-Inv-Dup	624	Repeat	Repeat_39494
23151	2	2648789..2649409	UnBal-Inv-Dup	620	Repeat	Repeat_40108
23712	2	383807..384764	Bal-Inv-Framt	957	Repeat	Repeat_38367, Repeat_38368
24146	2	4837884..4838548	UnBal-Inv-Dup	664	Repeat	Repeat_41631
26318	3	1013398..1014058	UnBal-Inv-Dup	660	Repeat	Repeat_57671
29142	3	18696965..18697347	UnBal-Inv-Dup	382	Repeat	Repeat_70838, Repeat_70839
29263	3	19066495..19066675	UnBal-Large-Dup	180	Repeat	Repeat_71125
29263	3	19068151..19068360	UnBal-Large-Dup	209	Repeat	Repeat_71125
32395	3	8050690..8051662	Bal-Inv-Framt	972	Repeat	Repeat_62915
43139	5	128216..128814	UnBal-Inv-Dup	598	Repeat	Repeat_94279
46422	5	6900639..6900801	UnBal-Trans	162	Repeat	Repeat_99387, Repeat_99388
46422	8	11283205..11283711	UnBal-Trans	506	Repeat	Repeat_151873, Repeat_151874
52028	6	2620470..2620776	UnBal-Trans	306	Repeat	Repeat_108508, Repeat_108509
52028	8	11283214..11283719	UnBal-Trans	505	Repeat	Repeat_151873, Repeat_151874
58484	7	4749087..4750073	Bal-Inv-Framt	986	Repeat	Repeat_130958
58485	7	4749430..4750258	Bal-Inv-Framt	828	Repeat	Repeat_130958
63963	8	7122023..7122827	Bal-Inv-Framt	804	mRNA	ppa026667m
64422	8	9086244..9087200	Bal-Inv-Framt	956	Repeat	Repeat_150549
**'F8,1-42’**						
**ID**	**Scaffold**	**Coordinates**	**SV Type**	**Length**	**Sequence**	**Gen or Repeat**
20993	2	10443560..10444206	Bal-Inv-Framt	646	-	-
21986	2	12156442..12157007	UnBal-Inv-Dup	565	Repeat	Repeat_46629, Repeat_46630
24536	2	16606936..16607425	UnBal-Inv-Dup	489	-	-
29055	2	2650281..2650575	UnBal-Inv-Trans	294	Repeat	Repeat_49991, Repeat_49992
29055	3	15046335..15046888	UnBal-Inv-Trans	553	Gene	ppa020237m.g
30173	2	4307001..4307685	UnBal-Inv-Dup	684	Repeat	Repeat_41315, Repeat_41316, Repeat_41317
33929	3	10480044..10480270	UnBal-Inv-Dup	226	Gene	ppa011613m.g
37571	3	19066494..19066675	UnBal-Large-Dup	181	-	-
37571	3	19068151..19068359	UnBal-Large-Dup	208	Repeat	Repeat_71125
46467	4	19153499..19153637	UnBal-Inv-Dup	138	Repeat	Repeat_86571
55460	5	10569336..10569979	UnBal-Inv-Dup	643	EST	EST217 [GenBank ID: FE969391.1]
55461	5	10569391..10570047	UnBal-Inv-Dup	656	EST	EST217 [GenBank ID: FE969391.1]
65545	6	19832212..19832895	UnBal-Inv-Dup	683	Repeat	Repeat_121473, Repeat_121474, Repeat_121475
77074	7	4761867..4762779	Bal-Inv-Framt	912	Repeat	Repeat_130964
77412	7	5482889..5483887	Bal-Inv-Framt	998	EST	HPL-01-A08 [GenBank: DN552811.1]
84240	8	5353089..5353931	Bal-Inv-Framt	842	Repeat	Repeat_147771
**'Georgia Belle’**						
**ID**	**Scaffold**	**Coordinates**	**SV Type**	**Length**	**Sequence**	**Gen or Repeat**
2525	1	1390693..1391565	Bal-Inv-Framt	872	EST	PP_LEc0006H18f [GenBank ID: DW341826.1]
32059	2	191135..192115	Bal-Inv-Framt	980	EST	PP_LEc0012I17f [GenBank ID: DW342898.1]
33996	2	22282633..22282891	UnBal-Inv-Dup	258	Repeat	Repeat_53962
34581	2	23312824..23313409	UnBal-Inv-Dup	585	Repeat	Repeat_54614, Repeat_54615, Repeat_54616
37966	2	4837563..4838555	Bal-Inv-Framt	992	Repeat	Repeat_41631
49338	3	4508991..4509132	UnBal-Inv-Trans	141	Repeat	Repeat_60164
49338	7	1525434..1525564	UnBal-Inv-Trans	130	Repeat	Repeat_128579
57742	4	19154182..19154816	UnBal-Inv-Dup	634	EST	AJ873513 [GenBank ID: AJ873513.1]
69825	5	10568959..10570034	Bal-Inv-Framt	1075	EST	EST217 [GenBank ID: FE969391.1]
69826	5	10569191..10570123	Bal-Inv-Framt	932	EST	EST217 [GenBank ID: FE969391.1]
76451	5	6900036..6900768	UnBal-Inv-Dup	732	Repeat	Repeat_99387, Repeat_99388
95603	7	22382739..22383456	UnBal-Inv-Dup	717	Repeat	Repeat_143336
95633	7	22436698..22437437	Bal-Inv-Framt	739	Repeat	Repeat_143367
96867	7	4749469..4750167	UnBal-Inv-Dup	698	Repeat	Repeat_130958

*ID* identification number for each structural variant, *SV Type* Structural variant type, which includes *UnBal-Inv-Dup* Unbalanced Inverted Duplication, *Bal-Inv-Trans* Balanced Inverted Translocation, *Bal-Inv-Framt* Inversion of a genomic fragment, defined by balanced signatures, *UnBal-Large-Dup* Unbalanced large Duplication, *UnBal-Trans* Unbalanced Translocation, *Sequence* type of functional sequence, *Length* number of nucleotides rearranged in the sequence.

Structural Variants (SV) exhibit a different pattern than the small variants. A global comparison of SV showed that 258 structural variations with respect to the 'Lovell’ sequence were shared by the three genotypes. Among these genotypes, 329 structural variations occur with respect to the peach reference genome sequence, of which 292 are inter-chromosomal and 37 are intra-chromosomal. Inverted translocations (172) are the most frequent variation, followed by inversions and duplications.

**Figure 2:**
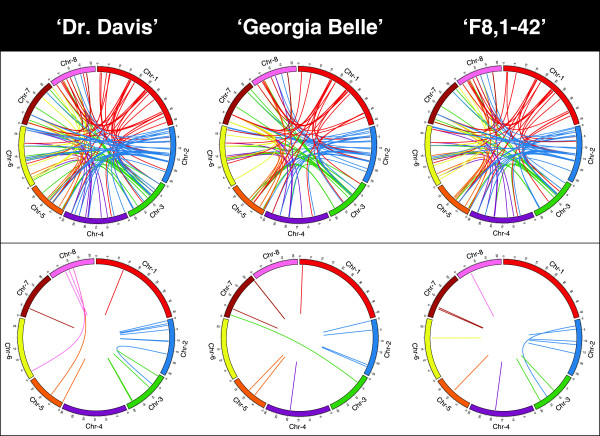
**Visual comparison of the structural variants for three peach cultivars using Circos graphs.** The variants were obtained through comparisons with the 'Lovell’ Peach Genome Reference Sequence ('Lovell’, upper row) and with the exclusive structural variants per genotype (lower row). Non-connected lines correspond to intra-chromosomal variations. Color of lines corresponds to the source chromosome as defined by the 'Lovell’ reference.

The number of exclusive SV in 'Dr. Davis’ was 285, 169 in 'F8,1-42’, and 151 in 'Georgia Belle’ (Figure 2). The number of exclusive SV with a high significance score per genotype longer than 100 nucleotides was 19 for 'Dr. Davis’ (detected by SVDetect release 0.8a). 'F8,1-42’ exhibited 14 structural variations, while 'Georgia Belle’ exhibited 13 (Figure 2, lower panel). Among the three genotypes, the most common types of SV were the unbalanced inverted duplications, or balanced inversions of genomic fragments. 'Dr. Davis’ exhibited one balanced inverted translocation and two unbalanced translocations, which occurred from the first third of chromosomes 5 and 6 to the middle part of chromosome 8. 'F8,1-42’ exhibited one unbalanced inverted translocation occurring between the first third of chromosome 2 and going to the middle part of chromosome 3, and one large unbalanced duplication in the terminal part of chromosome 3. 'Georgia Belle’ exhibited one unbalanced inverted translocation (details in Table 5) between the first fourth of chromosome 3 to the top of chromosome 7.

A search for genes within SV regions showed that, in 'Dr. Davis’, just two SV fell in regions with annotated transcripts in the genome annotation of the peach genome sequence reference: the gen ppb020139m.g and the mRNA ppa026667. The remaining SV fell in regions annotated with sequence repeats. A balanced inversion of a genomic fragment (Bal-Inv-Framt) with ID 63,963 in scaffold 8 is located at the gene ppa026667m. It is an mRNA, without a functional annotation. 'F8,1-42’ exhibits two SV within genic regions; a reciprocal translocation that affects the region of the Repeat_49992 in scaffold 2 and the region of the gen ppa020237m.g in scaffold 3, in addition to an inversion within the gen ppa011614m.g in scaffold 3. Three SV (two in scaffold 5 and one in scaffold 7) overlap with Expressed Sequence Tags (ESTs).

'Georgia Belle’ had no SV overlap with a genic region, and five SV (in scaffolds 1, 2, 4 and 5) overlapped with the PP_LEc0006H18f [GenBank ID: DW341826.1], PP_LEc0012I17f [GenBank ID: DW342898.1], ESTs AJ873513 [GenBank ID: AJ873513.1] and EST217 [GenBank ID: FE969391.1] (Additional details in Table 5).

### Genome-wide comparison

A conservation matrix was obtained (Table 6) from the genome-wide comparison through the pairwise alignment of 'Lovell’ reference genome sequence and the three genotypes studied. Values of zero indicate complete genome conservation between a pair of genome sequences, while values greater than zero imply some degree of divergence between genome sequences (negative values are not expected), with the value of one denoting complete divergence between a pair of sequences.

**Table 6:** **Genome conservation matrix among the three genotypes and the peach genome reference sequence**

	'Lovell’	'Georgia Belle’	'Dr. Davis’	'F8,1-42’
**'Lovell’**	0	0.0264	0.0167	0.0430
**'Georgia Belle’**	-	0	0.0268	0.0429
**'Dr. Davis’**	-	-	0	0.0405
**'F8,1-42’**	-	-	-	0

The analysis, performed using Mauve 2.3.1, identified 'F8,1-42’ as the most divergent genotype with respect to the 'Lovell’ reference (0.0430). 'Georgia Belle’ was intermediate (0.0264), while the least divergent was 'Dr. Davis’ (0.0167). The divergence between 'F8,1-42’ and 'Georgia Belle’ (0.0429) was comparable to that between 'Lovell’ and 'F8,1-42’, and similar to that exhibited between 'F8,1-42’ and 'Dr. Davis’ (0.0405). The divergence between the two peach cultivars was 0.0268, which was comparable to divergence between 'Lovell’ and 'Georgia Belle’. The analysis also determined that the three genotypes exhibit a GC-content of 37.6%.

## Discussion

Small variants and structural variants represent different types of genomic variation. While natural selection acts on both types, crop breeding targets primarily small variants, as their inheritance patterns are better understood and therefore, more efficiently manipulated, and because small variants code for single functional changes (amino acid and protein changes). Most crop breeding programs target small incremental changes, while structural variation is manifested as large disruptive changes, including possible sterility as result of genome mismatch. An improved understanding of the process through which structural variants occur, their locations, and their effects on phenotype expression, is now possible through advanced genomic methods.

### Small variants

SNP ratios (SNP/bp) observed in this study, differ from previous results observed in other crop plants, which typically occur in a range between 1/100 and 1/300 bp [37]. The SNP/bp ratio also differs among genotypes with respect to the clonal age of the peach cultivars. The heirloom melting flesh cultivar 'Georgia Belle’ (originating before 1870) presented the largest SNP/bp ratio (1/391), agreeing with results of Aranzana et al. [38] showing the highest heterozygozity for this type of cultivar. In contrast, 'Dr. Davis’, which was selected in 1979 and patented in 1982 [39, 40], exhibited a ratio of 1/633, suggesting that modern cultivars tend towards a more homogeneous genomic state, with its associated higher homozygosity. This trend would be an expected consequence of the self-fruitfulness of this species combined with its narrow genetic base, since most important European and North American cultivars have been derived from as few as six Chinese founder genotypes [41]. Both factors promote inbreeding, which leads to homozygosity.

'Georgia Belle’, which is a progeny of 'Chinese Cling’, one of the founder genotypes for modern cultivated peaches, is a melting flesh cultivar, whereas 'Dr. Davis’ is non-melting. Aranzana et al. [38] divided peach cultivars into three main groups based on fruit type rather than geographical distribution [42]. They found that melting flesh cultivars tend to be more heterozygous and probably represent the predominant first domesticated peach types.

'F8,1-42’ exhibited a SNP ratio of 1/415. Selection 'F8,1-42’ represents a more exotic genotype, since the related species *Prunus dulcis* (Mill.) D.A.Webb (almond) was used as the seed parent in one cross in its lineage (see Additional file 4) [43]. The SNP variant event ratio was closer to that for 'Georgia Belle’ than for 'Dr. Davis’. The genome conservation distance matrix among the four sequences suggests that the almond background in 'F8,1-42’ influences the zygosity of this selection as well as the divergence of the genome sequence relative to 'Lovell’, 'Georgia Belle’, and 'Dr. Davis’.

Earlier studies of the introgression of almond to peach have shown that the rate of recombination between genomes is reduced [44]. Hence, long donor chromosome segments were maintained, resulting in linkage drag. This may be responsible for the wide range in the variants, as well as the change ratios (variant/bp) per scaffold in 'F8,1.42’ (from 1 change every 122 bases to 1 in 1111 bases). Consequently, further backcrossing to peach is desirable to add and fix desired combinations into breeding selections. Interestingly, 'F8,1-42’ exhibits a unique non-melting, freestone phenotype which has not been previously reported in peaches [45], suggesting that the expression of this unique phenotype is a result of unique recombinations of almond and peach genetic material [46].

The differences in the change rates among chromosomes and within chromosomes or scaffolds is, in part, a result of the pattern of crossovers along chromosomes, which is influenced by the length of the chromosome [47] and position on the chromosome [48], as well as genome compatibility in interspecific crosses. Scaffold 2 in all three genotypes exhibited the highest change rate, even though it is not the largest chromosome. The ranking from longest to shortest based on sequencing in the peach reference genome sequence is: scaffold 1, scaffold 4, scaffold 6, scaffold 2, scaffold 7, scaffold 3, scaffold 8 and scaffold 5.

The high rate of variation for chromosome 2 may be a result of the higher number of recombination hotspots, as has been reported by Nachman in the case of humans [49]. Scaffold 2 has been reported to carry important quantitative trait loci (QTL) for fruit, including ripening time, skin color, soluble solids content, and diameter [50], which are important targets of selection. More recombination does not necessarily represent a source of new alleles, since recombination hotspots often occur in intergenic regions in plants [51, 52], and their distribution along the chromosome is influenced by several factors, including proximity to the centromere, gene density, and GC content [53]. A better understanding of the distribution of these hotspots will lead to better modeling of the inheritance and conformation of linkage blocks. Relatively large linkage blocks are anticipated in peach because of the low linkage disequilibrium decay in the species, which ranges from ~6 cM (2524–2644 Kb) in Chinese landraces [42] to 13 to 15 cM (5460–6600 Kb) in commercial cultivars [38].

Scaffold 4 has been reported to carry QTLs for blooming time, ripening time, and glucose/fructose content, as well as the major genes for flesh adhesion (*F*) (clingstone/freestone) and flesh texture (*M*) (melting/non-melting) [17], which are discriminator traits for the three genotypes studied here, as well as important targets of selection in the Processing Peach Breeding Program at UC Davis. Also, scaffold 4 is the third longest scaffold in peach, and has exhibited one change every 330 bp in 'Dr. Davis’, one for every 352 bp in 'F8,1-42’, and one for every 505 bp in 'Georgia Belle’ (Figure 1). High rates of variation were exhibited in the terminal sections of the scaffold in the three genotypes, which coincide with identified QTLs for freestone-melting flesh, mealiness, and flesh bleeding in two mapping populations obtained through two crosses using the three genotypes studied here ('Dr. Davis’ used as seed parent in both crosses) [54]. The variations in the genome-wide change rate and scaffold change rate in the three genotypes studied here do not represent some systematic change, but such variations are likely to be due to random variation. However, if different chromosomes have different distributions of non-coding DNA, such difference in non-coding DNA distribution could imply some rate change bias.

Most of the genomic variations would be expected to occur within non-coding regions, thus avoiding changes to transcribed proteins [55]. A relatively low numbers of high impact variants (splice site acceptors, splice site donors, start lost codons, frame shifts, stop gained codons, and stop lost codons) were observed. These variants can alter the amino acid transcript or the length of the ORF and directly impact the structure of the protein. These results were expected since one of the DNA functions is to prevent disruptive changes, which can compromise the integrity of the organism.

The proportion of silent changes (around 39%) and missense modifications (around 58%) among the three genotypes is relevant since the former are considered as evolutionarily neutral (however, these silent changes can affect the structure and function of the resultant protein, see [56]) and the latter are not. Our results support that, from an evolutionary perspective, the proportion of missense and silent modifications, as well as the ratio between these modifications, indicate a strong effect of artificial selection on the peach genome over the last 100 years of cultivar breeding.

The observed genome-wide missense/silent modifications ratios are consistent with the theory that loci under the action of selection present higher ratios of missense/silent modifications than do those under less or nil selection pressure. Thus, if the whole genome is considered as a whole transcribe-able locus, the heirloom cultivar 'Georgia Belle’ exhibited a value of 1.4481, while the modern 'Dr. Davis’ exhibited a value of 1.5262. Selection 'F8,1-42’, with its introgression of genetic material from almond, exhibited a value of 1.4347, which was more similar to the more diverse heirloom cultivar. While these analyses ultimately have to be performed on specific loci (genes or candidate genes, preferably those with agronomic value) they provide initial insights into the ways that artificial selection has configured the peach genome including targets of selection, methods of selection and timing, as has been suggested by Aranzana et al. [41] and Verde et al. [4].

The transition-transversion ratio (Ts:Tv) is around 3.0, which is consistent with the Ts:Tv ratio of 3.0988 from SNPs mapped in closely related peach genotypes reported by Martinez-Garcia et al. [57]. Ts:TV ratios in Non-long Terminal Repeat (Non-LTR) retrotransposon sequences have been estimated as 3.9, 3.6, 1.9, 1.6, and 2.5 for plants such as maize, alfalfa (*Medicago sativa* L.), eikorn wheat (*Triticum monococcum* L.), barley (*Hordeum vulgare* L.) and plants from the genus *Lotus*, respectively [58]. Information about Ts:Tv ratios in whole genome sequences from other peach relatives, or even other crops, is scarce. The transition-transversion ratio is commonly used for phylogenetic tree reconstruction, divergence time estimation, as well as a better understanding of the mechanisms of molecular evolution [59, 60]. It is a theoretical estimator of mutation rates and evolutionary divergence, which is not directly related to observed rates of change at the phenotypic level [61].

'F8,1-42’ and 'Georgia Belle’ exhibited the same most common amino acid substitutions, Alanine to Valine, Valine to Isoleucine and Alanine to Tyrosine. Nucleotide and amino acid substitutions have been shown to affect important agronomic traits. Barry et al. [62] identified two mutations involved in the degradation of green color in tomato, which can be traced to two specific amino acid substitutions. Previous studies in peach have shown a Quantitative Trait Nucleotide (QTN) located on chromosome 4 to be involved in chilling injury, in particular mealiness [57]. The understanding of nucleotide and amino acid substitutions can therefore facilitate the characterization of metabolic pathways and improvements in phenotyping through the identification of the relevant biochemical changes affecting structure or the availability of substrates.

### Structural variants

The peach genome is approximately 227.3 Mb long, and has approximately 62.3Mb (27.4%) of repeats (see [63]); so the effective coding sequence of peach is approximately 165 Mb in length. With 27,852 genes annotated ([4] and see [64]), the average length of a gene in peach is approximately 5924 bp. Thus, if a balanced inversion of a genomic fragment occurred in a genic region, it would constitute a sizable structural change, which could compromise the function of associated genes or prevent recombination in that region. In this particular case, the structural variant with ID 69,825 occurs in scaffold 5, within a reported EST (GenBank ID FE969391.1) described as a protein of unknown function [65].

The majority of the exclusive variants in our analysis were found within repeats. Thus, 'Dr. Davis’ exhibited an unbalanced inverted duplication (UnBal-Inv-Dup) within the gen ppb020139m.g in scaffold 1 (variant ID 1495, Table 5), which is associated with the cytochrome C assembly protein family, in homologous *Arabidopsis thaliana* L. and rice sequences.

Construction of a complete reliable functional annotation for peach has not been completed [9]. An initial annotation was done several years ago (see [66]); however, there are gaps and inconsistencies such as the unbalanced inverted translocation (UnBal-Inv-Trans) occurring between scaffolds 2 and 3, associated with a non-plant functional annotation for the human Fanconi anemia pathway. The, Kegg Orthology (entry K10891) for this annotation is “a rare genetic disorder characterized by aplastic anemia, greater susceptibility to cancer/leukemia as well as cellular hypersensitivity to DNA crosslinking agents, such as cisplatin” [67].

An UnBal-Inv-Dup (ID 33,929) was present in the first exon of gene ppa011613m.g, which appears related to Ribosomal protein L13, controlling the structural constituents of the ribosome. Two UnBal-Inv-Dup and one Bal-Inv-Framt overlapping within two ESTs, (one of them being the same EST described above in 'Georgia Belle’,) occurred twice in 'F8,1-42’. The Bal-Inv-Framt (ID 77,412) overlapped with the EST HPL-01-A08 (GenBank: DN552811.1 from a Plum Pox Virus (PPV) study [68], in which this particular EST was obtained from non-infected 'Baby Gold #5’ cultivar leaf tissue).

The distribution of variants observed in chromosomes 4 and 8 of 'F8,1-42’ (Figure 1) suggested that SV has occurred at the terminal portions of the chromosome. Thus, on chromosome 4, seven translocations (Trans) and inverted translocations (Inv-Trans) between the nucleotides 19,153,501 and 27,502,845, in addition to four inverted duplications (Inv-Dup) have occurred (details in Additional file 5, sheet F8_Exclusive). Chromosome 8 in 'F8,1-42’ exhibited seven translocation and inverted translocations events between the nucleotides 11,283,140 and 17,453,927. It has been reported that QTLs for chilling and heat requirement are located within the middle and terminal portion of chromosome 8 [69]; therefore, the SV reported in 'F8,1.42’ for this chromosome would have implication in altering characteristics such as blooming date (BD) or maturation time (MT). For the three genotypes studied, the number of Julian days for BD and MT are different among genotypes by 10 to 15 days, being the earliest for 'Georgia Belle’, followed by 'Dr. Davis’, and 'F8,1-42’ (latest flowering). These SV are not exclusive to 'F8,1-42’, since some are shared with least one other genotype (mostly 'Dr. Davis’).

A set of 62 SV (of 292), on chromosome 8, was shared by the three genotypes, and those SV were different from that of 'Lovell’, which suggests that this specific chromosome has undergone a severe rearrangement. In the case of 'F8,1-42’ rearrangement effects may be magnified as a result of almond genetic material introgression. However, this restructuring had also taken place (to a limited extent) in the other genotypes, as seen in by Jauregui et al. [70] in F_2_ progeny between an almond and peach with introgression of *Prunus davidiana* (Carrière) Franch in upstream generations, indicating that this chromosome is under constant restructuring in peaches. Restructuring may be occurring as a result of the mode of evolution shaping the *Prunus* genome, as it is hypothesized that the ancestral genome of Rosaceae had nine chromosomes [71], and that chromosome 8 in *Prunus* may have resulted from a fission event in the Rosaceae ancestral chromosome A1, when the shortest portion formed chromosome 8, and the fusion of the largest portion of A1 and the whole A2 formed chromosome 1 [72]. Similarly, chromosome 4 was formed from the larger portion of an A9 fission event, while the smaller A9 portion fused with A8 to form chromosome 6 [72]. Interestingly, chromosome 4 carries genes relevant to the fruit phenotypic differences among the three genotypes in this study (particularly genes *F* and *M* mentioned above, which are located within the range of high frequency of variation); but chromosome 8 in *Prunus* is recognized as a chromosome with little evidence for the maintenance of simply inherited (and critical) genes [73] or QTLs [74] responsible for the anthropocentric discrimination of useful agronomic traits used for subsequent selection of peaches during domestication and current breeding.

'Georgia Belle’, in addition to the EST mentioned above, displayed exclusive structural variants (inversions) overlapping with ESTs: PP_LEc0006H18f (GenBank ID: DW341826.1) and PP_LEc0012I17f (GenBank ID: DW342898.1) [75]. The EST AJ873513.1 (GenBank ID: AJ873513.1) has been identified in mesocarp with epidermis tissues at 30 days after bloom in studies of the early stages of fruit development in the peach cultivar 'Fantasia’ (unpublished data [76]).

An estimation of divergence among genotypes provides an overview of whole genome differences. Thus, the divergence between a complete homozygous genome ('Lovell’) and an heirloom cultivar ('Georgia Belle’) is comparable to that exhibited by a genotype of peach with introgressed material from almond ('F8,1-42’). This finding suggests that introgression from almond and subsequent backcrosses with conventional peach genotypes promotes genome heterogeneity similar to that exhibited by the direct progeny of the peach founder genotype 'Chinese Cling’. The divergence between 'Lovell’ and 'Dr. Davis’ supports the assertion that modern cultivars of peach tend to be genomically homogeneous and, thus, tend to be more homozygous. The genomic divergence between 'Georgia Belle’ and 'Dr. Davis’ possesses relevance in terms of fruit characteristics, since the genotypes are completely opposite. 'Georgia Belle’ is a cultivar selected for fresh consumption of the fruit, since the fruits are freestone, melting and white, while 'Dr. Davis’ is a cultivar for the processing industry (e.g. canning and baby food production), with fruits that are clingstone, non-melting and yellow. However, 'Dr. Davis’ and 'Lovell’ fruits are phenotypically distinct only with respect to the detachment of the endocarp from the mesocarp, since the fruits are clingstone and freestone, respectively, and they exhibit the least divergence among the four genotypes.

Our results were consistent with previous discoveries from other crops. In watermelon [*Citrullus lanatus* (Thunb.) Matsum. & Nakai, Cucurbitaceae], genome heterogeneity has been observed in genomic regions affected by the domestication process, such as disease-resistance genes [77]. In the case of soybean (Fabaceae), a comparison between wild and cultivated soybeans showed long Linkage Disequilibrium blocks in cultivated soybeans, which may result from a combination of the lower genetic diversity given by the domestication bottleneck, low frequency of genetic recombination, and self-fertilization [78]. Similar processes can also be occurring in peach [13, 14].

Several resequencing projects of genomes at the intra-specific level (cultivar founders, breeding lines, cultivars, hybrids) have been carried out to understand genomic heterogeneity [33, 77–82]. In tomato (*Solanum lycopersicum* L.), the model species for the evolution of species possessing fleshy fruits [83], more than 150 genotypes are being resequenced in the largest resequencing project until now for a crop species [84], The discoveries from this kind of project will have significant relevance for their application in various biological systems of several agricultural crop species. However, researchers should be cautious when extrapolating results, since differences in biology, life history, crop production systems, etc. may result in comparisons/correlations that are not appropriate. For example, peach is a vegetatively propagated species (cloned) and intra-cultivar genome heterogeneity is not an issue, while for soybeans, a sexually generated crop, it is a consideration [33]. The extrapolation of results from closely related species should be done cautiously. For example, although apple (*Malus × domestica* Borkh., Rosaceae) is a closely related species to peach and vegetatively propagated, apple’s domestication history is totally different [85]. Hence, the context in which each biological system has evolved is relevant when making decisions about which discoveries can be extrapolated.

Our findings suggest that identification of genomic variants may be particularly important in breeding programs incorporating interspecies germplasm to expand the genetic base. A more accurate characterization of the structural variants identified could facilitate “smart breeding”, as suggested by McCouch et al. [86], thus facilitating the recycling of genes that domestication and associated artificial selection had left behind. A useful tool is the genome conservation matrix, which estimates the extent of the genetic-genomic difference between one genotype and another through measurement of their divergence-conservation distance. Thus, the genome conservation matrix “expresses the conservation of both sequence and gene content between two genomes” [36].

This study, to the authors’ knowledge, is the first to use the measurement of conservation-divergence to compare three phenotypically distinct peach genotypes, two commercial peaches, and a peach with almond in its pedigree. Although this measurement may be biased as a result of the assumption of same gene content (an unbiased assessment would require a *de novo* genome sequence, structural and functional annotations per genotype) and the absence of a comparison with the almond genome sequence (not yet completed). However, given the current status of and the trends for high-throughput sequencing and the comparison of individual genomes [87], future reports with enhanced accuracy and specific trait targets will likely be published.

## Conclusions

We combined Illumina/Solexa and Roche 454 sequences to evaluate the genome heterogeneity in three peach genotypes using the doubled haploid cultivar 'Lovell’ as reference sequence. We counted the number of small variants and structural variants among these genotypes and we also estimated the divergence between each genome with the peach reference genome. The main objective was to try to understand the quantitative differences in peach genome sequences and improve the knowledge about the relationship of phenotype and genome features through the application of bioinformatic procedures.

The heterogeneity among the genomes of three peach genotypes was analyzed to characterize and quantify genomic variants. Further analysis showed that the heirloom cultivar 'Georgia Belle’ and the almond by peach introgression breeding line F8,1-42’ are more heterogeneous than is the modern cultivar 'Dr. Davis’, when compared with the 'Lovell’ peach reference genome. The differences in heterogeneity per peach genotype are reflected in the number of variants, the types of variants, and the impacts of those variants on the transcribe-table and non-transcribe-table portions of each genotype analyzed.

The pair-wise comparison of consensus genome sequences with 'Lovell’ showed that 'F8,1-42’ and 'Georgia Belle’ are more divergent compared to 'Dr. Davis’ and 'Lovell’. The results suggest that progenies close to peach founder genotypes conserve more heterogeneity than modern cultivars do, and that the introgression of genetic material from related species can promote genomic heterogeneity in modern breeding lines.

The study of genomic variants is useful for the elucidation of genetic control of pomological traits, the characterization of metabolic pathways and the modeling of the inheritance of complex traits, and thus can lead to improved protocols for phenotyping in research and breeding.

## Methods

### Plant materials

'Georgia Belle’ (also called 'Belle of Georgia’ [88]) is a freestone peach (the endocarp detaches freely from the mesocarp) with white flesh obtained no later than 1870 on the East Coast of the US. It exhibits melting flesh (losing of firmness and structure, for an accurate description see [89]), a high acid/sugar ratio, and is prone to flesh mealiness and significant browning. This cultivar is a progeny from an open pollination of a tree of the cultivar 'Chinese Cling’; however, other studies suggest the cultivar 'Late Crawford’ is the male parent [88].

'Dr. Davis’ is a clingstone peach (the endocarp does not detach freely from the mesocarp) with yellow flesh, exhibiting non-melting flesh and bland-flavor, with a non-mealy flesh showing only slight oxidative-browning. It is considered a quality reference for canning peach cultivars [39]. It was patented in 1982 (PP4861) and is the result of a cross between the selections D25-9E and G40-5E in the UC Davis breeding program.

'F8,1-42’ is an advanced breeding line with an exotic genetic background including an almond introgression ('Nonpareil’) and several processing peach cultivars (e.g. 'Jungerman’ and 'Everts’) in its lineage. Therefore, it is considered to be an exotic breeding accession, although it is distinctly peach for all fruit and tree phenotypes. It has an unusual phenotype combination, as it has non-melting flesh at maturity, comparable to the standard canning clingstone peach cultivars. Unlike standard canning clingstone peach cultivars; however, it is a freestone, non-melting cultivar. Consequently, F8,1-42 is the breeding line closest to the much desired Non-melting-Freestone cultivar, even though it appears to possesses the standard Non-melting-Clingstone endopolygalacturonase (*endoPG*) *f1* allelic genotype [46].

### Methods

For this study, the binary alignment mapped (BAM) files generated from the study of Ahmad et al. [25] were used to generate Simple Alignment Map (SAM) and, subsequently, Variant Filter Calling (VCF) files through the use the routine mpileup in the software SAMtools [90]. The alignment files were developed from the combined Illumina/Solexa and Roche 454 sequences for 'Dr. Davis’ and 'F8,1-42’, and exclusively Illumina/Solexa for 'Georgia Belle’. The alignments were performed with the Burrows-Wheeler Aligner (BWA) tool [91, 92] against the peach reference genome 'Lovell’ (available at [64]). As given by Ahmad et al., aligned positions for 'Dr. Davis’, 'F8,1-42’ and 'Georgia Belle’ were calculated to be 94.7%, 92.0% and 93.7%, respectively. Additionally, consensus genome sequences were generated per genotype through the application of the routine: samtools mpileup -uf ref.fa aln.bam | bcftools view -cg - | vcfutils.pl vcf2fq > cns.fq to each BAM file, resulting in three files in FASTA format of 230.1 MB each.

The quantification, estimation of general statistics, distribution, and prediction of effects on the genomic variants were performed with the software SnpEff 3.0c [93], and are available at the developer’s web page [94]. This software is a bioinformatics tool that annotates the variants (SNPs, insertions, deletions, and multiple nucleotide polymorphisms) and calculates the effects they produce on known genes present in the annotation of the reference genome sequence through an algorithm based on interval trees, which is implemented in the Java programming language.

A SnpEff predictor database file in binary format (.bin) was created to locate each SNP within annotated transcripts or intronic regions. This predictor database is available through SnpEff, and it is based on the 'peach v1.0 genome’ sequence. Annotation of the peach v1.0 is available at Genome Database for Rosaceae (GDR) [64], which was generated by gene models based on homology prediction using information publically available from several organisms. The default parameters of SnpEff ver 3.0c were used to generate the predictor database and perform the Variant Effect Analysis of the three genotypes of peach in annotated transcripts within the 5000 bases of the upstream and downstream portions of the Open Reading Frames (ORF). Both HTML and text output files were generated from SnpEff. The output included the position of the SNP on the scaffold, the reference nucleotide, the changed nucleotide, whether it was a transition or a transversion, the transitions/tranversions ratio (Ts/Tv), warnings, the gene ID, the gene name, the biotype, the transcript ID, the exon ID, the exon rank effect, the amino acid change (old aa/new aa), old codon/new codon, the number of effects, the effects by functional class, the missense/silent ratio, the codon number [based on the coding sequence (CDS)], and the CDS size.

SVDetect release 0.8a [95] was used for the detection of structural variants. This program is specifically designed to identify genomic structural variations through sliding-window and clustering strategies by processing sorted BAM or SAM files resulting from the alignment of the whole sequences for 'Dr. Davis’, 'F8,1-42’ and 'Georgia Belle’ against 'Lovell’. Each alignment file was processed, using a read length of 84, window size of 832 in 'Dr. Davis’, 840 for 'F8,1-42’, and 915 for 'Georgia Belle’. The step length values were 208, 210, and 229, respectively. The values for window size and step size were calculated by running the script BAM_preprocessingPairs.pl (included in SVDetect) per genotype. The script outputs the values for mu_length and sigma_length parameters. Once the values were set for each genotype, all the structural variants (inter and intra chromosomal, as well as balanced and unbalanced) were identified and quantified to convert the output to a graphical form through the visualization tool Circos 0.6.2 [96].

Mauve 2.3.1 [97] [progressiveMauve (multiple genome alignment) using the default settings and the assumption of collinear genomes for the four sequences] was used for the pair-wise comparison among the three consensus genome sequences of the three genotypes previously generated through SAMtools and the peach genome reference genome 'Lovell’.

## Authors’ information

**JFR** is a PhD Candidate in the field of plant genetics and breeding. Currently working on the development and application of genomic resources for the breeding of peach and almond. Areas of interest are plant genetic resources, applied bioinformatics, quantitative genetics and the breeding of fruit tree crops.

**PJMG** Postdoctoral Associate at UC Davis, Department of Plant Sciences, in David Neale’s lab. His research focuses on genetic and comparative mapping, marker-assisted selection, breeding, population genetics and genome evolution in forest trees.

**DEP** Lecturer and Pomologist in the College of Agricultural and Environmental Sciences (AES). He is a plant geneticist, breeder with a research focus on fruit and nut germplasm diversity, genetic relationships, and tree breeding.

**CHC** Specialist and Pomologist, his research and extension program is focus on the postharvest biology and technology of fruits through the application of genomic techniques to identify gene(s) responsible for fruit sensory attributes (both desirable and undesirable), and investigating physiological disorders such as chilling injury.

**TMG** Professor and Breeder, his research focuses on the development of improved breeding lines and varieties of almond and processing peach through introgression of genetic material from other *Prunus* relatives to solve problems such as brown rot of clingstone peach, aflatoxin contamination of almond, and pollination efficacy in almond.

## Electronic supplementary material

Additional file 1: **Summary file of SnpEff output for 'Dr. Davis’.** SnpEff_DD.pdf: Summary of statistics of the output of SnpEff 3.0c for the variants present in the peach genotype 'Dr. Davis’ in portable document format (PDF). (PDF 757 KB)

Additional file 2: **Summary file of SnpEff output for 'F8,1-42’.** SnpEff_F8.pdf: Summary of statistics of the output of SnpEff 3.0c for the variants present in the peach genotype 'F8,1-42’ in portable document format (PDF). (PDF 806 KB)

Additional file 3: **Summary file of SnpEff output for 'Georgia Belle’.** SnpEff_GB.pdf: Summary of statistics of the output of SnpEff 3.0c for the variants present in the peach genotype 'Georgia Belle’ in portable document format (PDF). (PDF 796 KB)

Additional file 4: **Pedigree of the advanced breeding line 'F8,1-42’.** F8,1-42_Ped.pdf: 'F8,1-42’ has an exotic genetic background, including introgression of almond (*P. dulcis*) from the cultivar 'Nonpareil’ (pink box in the center) and several peach cultivars. This figure was generated through PediMap® version 1.2 [98]. (EPS 1 MB)

Additional file 5: **Summary of SV identified in the three peach genotypes.** SV_DD_F8_GB.xls: Summary of the intra and inter-chromosomal SV identified in exclusive or shared among the peach genotypes 'Dr. Davis’, 'F8,1-42’ and 'Georgia Belle’ in Microsoft Excel format (XLS). (XLS 268 KB)
