# Supplementary material for: Heterogeneity in the entire genome for three genotypes of peach [Prunus persica (L.) Batsch] as distinguished from sequence analysis of genomic variants
Source: BMC Genomics. 2013 Nov 1;14(1):750. doi: 10.1186/1471-2164-14-750 (PMC4046826; doi:10.1186/1471-2164-14-750)
Supplement: Supplementary file 1 — Additional file 1: Summary file of SnpEff output for 'Dr. Davis’. SnpEff_DD.pdf: Summary of statistics of the output of SnpEff 3.0c for the variants present in the peach genotype 'Dr. Davis’ in portable document format (PDF). (PDF 757 KB) [file 12864_2013_5471_MOESM1_ESM.pdf]

SnpEff: Variant analysis

Contents

Summary
Change rate by chromosome
Variants by type
Number of variants by impact
Number of variants by functional class
Number of variants by effect
Quality histogram
Coverage histogram
Base change table
Transition vs transversions (ts/tv)
Frequency of alleles
Codon change table
Amino acid change table
Chromosome change plots
Details by gene

Summary

Genome	ppersica139
Date	2012-09-03 21:34
SnpEff version	SnpEff 3.0c (build 2012-07-30), by Pablo Cingolani
Command line arguments	SnpEff ppersica139 -i vcf /Volumes/USB DISK/VCFs/DD.vcf -s /Volumes/USB DISK/VCFs/DD.html -v
Warnings	0
Number of lines (input file)	396,433
Number of variants (before filter)	399,649
Filter	
Number of variants filtered out	0
Number of not variants<br>(i.e. reference equals alternative)	0
Number of variants processed<br>(i.e. after filter and non-variants)	399,649
Number of known variants<br>(i.e. non-empty ID)	0 (0%)
Number of effects	630,488
Genome total length	227,252,106
Genome effective length	227,128,408
Change rate	1 change every 568 bases

Change rate details

Chromosome	Length	Changes	Change rate
1	46,877,626	89,357	524
2	26,807,724	67,505	397
3	22,025,550	21,525	1,023
4	30,528,727	60,371	505
5	18,502,877	33,904	545
6	28,902,582	32,778	881
7	22,790,193	58,020	392
8	21,829,753	17,208	1,268
Total	227,128,408	399,649	568

Number changes by type

Type	Total	Homo	Hetero
SNP	358,648	1,428	357,220
MNP	0	0	0
INS	19,148	6,756	12,392
DEL	21,853	6,995	14,858
MIXED	0	0	0
Interval	0	0	0
Total	399,649	15,179	384,470

Number of effects by impact

Type (alphabetical order)	Count	Percent
HIGH	1,691	0.268%
LOW	10,404	1.65%
MODERATE	15,797	2.506%
MODIFIER	602,596	95.576%

Number of effects by functional class

Type (alphabetical order)	Count	Percent
MISSENSE	15,618	59.289%
NONSENSE	491	1.864%
SILENT	10,233	38.847%

Missense / Silent ratio: 1.5262

Number of effects by type and region

Type

Type (alphabetical order)	Count	Percent
CODON_CHANGE_PLUS_CODON_DELETION	64	0.01%
CODON_CHANGE_PLUS_CODON_INSERTION	79	0.013%
CODON_DELETION	82	0.013%
CODON_INSERTION	35	0.006%
DOWNSTREAM	210,781	33.431%
FRAME_SHIFT	847	0.134%
INTERGENIC	108,303	17.178%
INTRON	47,897	7.597%
NON_SYNONYMOUS_CODING	15,537	2.464%
NON_SYNONYMOUS_START	2	0%
SPLICE_SITE_ACCEPTOR	121	0.019%
SPLICE_SITE_DONOR	144	0.023%
START_GAINED	169	0.027%
START_LOST	35	0.006%
STOP_GAINED	499	0.079%
STOP_LOST	45	0.007%
SYNONYMOUS_CODING	10,217	1.62%
SYNONYMOUS_STOP	16	0.003%
UPSTREAM	231,850	36.773%
UTR_3_PRIME	2,430	0.385%
UTR_5_PRIME	1,335	0.212%

Region

Type (alphabetical order)	Count	Percent
DOWNSTREAM	210,781	33.431%
EXON	27,458	4.355%
INTERGENIC	108,303	17.178%
INTRON	47,897	7.597%
SPLICE_SITE_ACCEPTOR	121	0.019%
SPLICE_SITE_DONOR	144	0.023%
UPSTREAM	231,850	36.773%
UTR_3_PRIME	2,430	0.385%
UTR_5_PRIME	1,504	0.239%

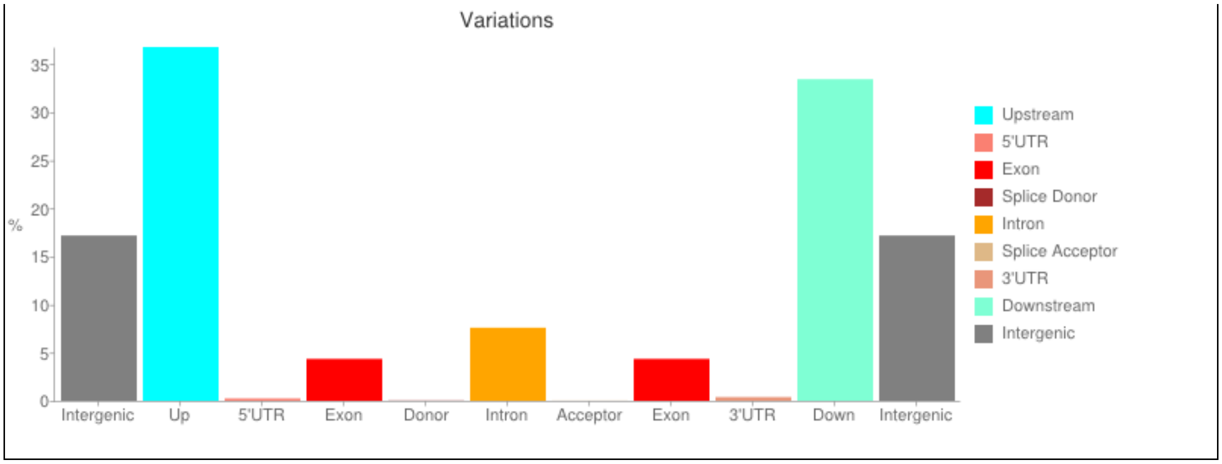

Quality:

Min	3
Max	227
Mean	85.614
Median	75
Standard deviation	63.336

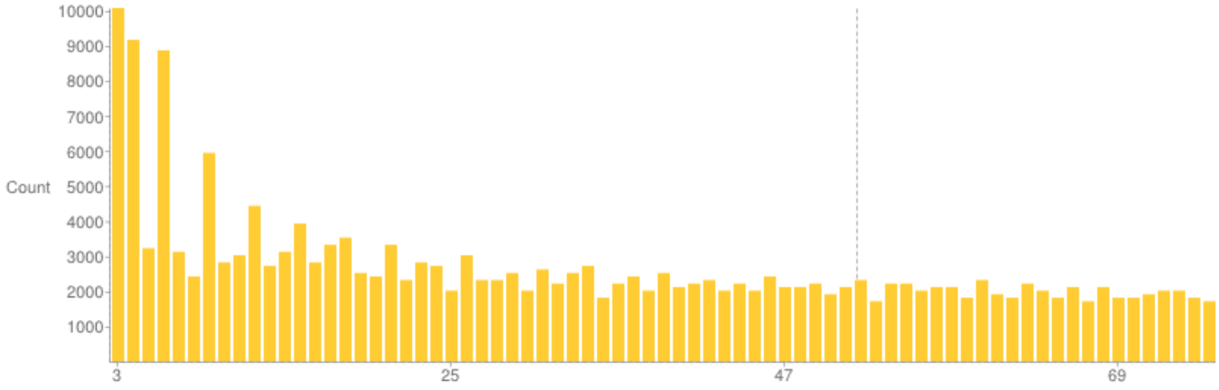

Coverage:

Min	1
Max	2,887
Mean	15.964
Median	13
Standard deviation	22.765

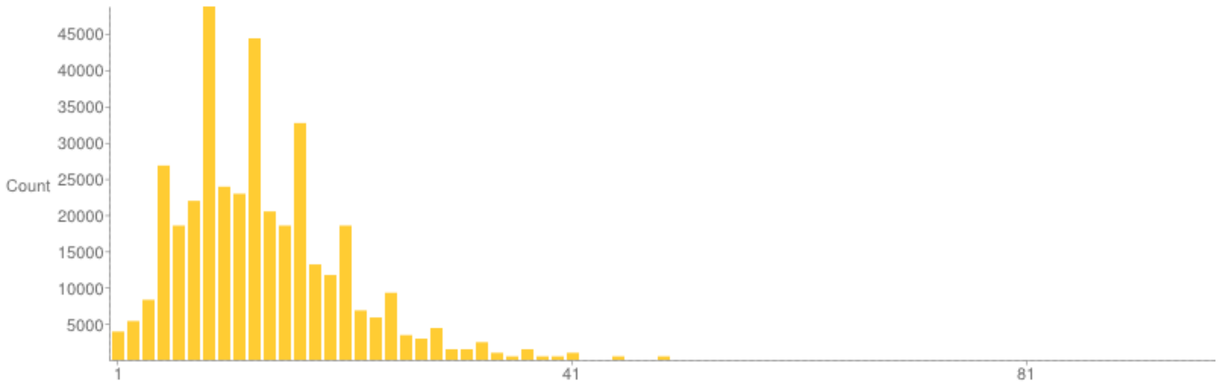

Insertions and deletions length:

Min	-49
Max	49
Mean	-0.528
Median	-1
Standard deviation	5.131

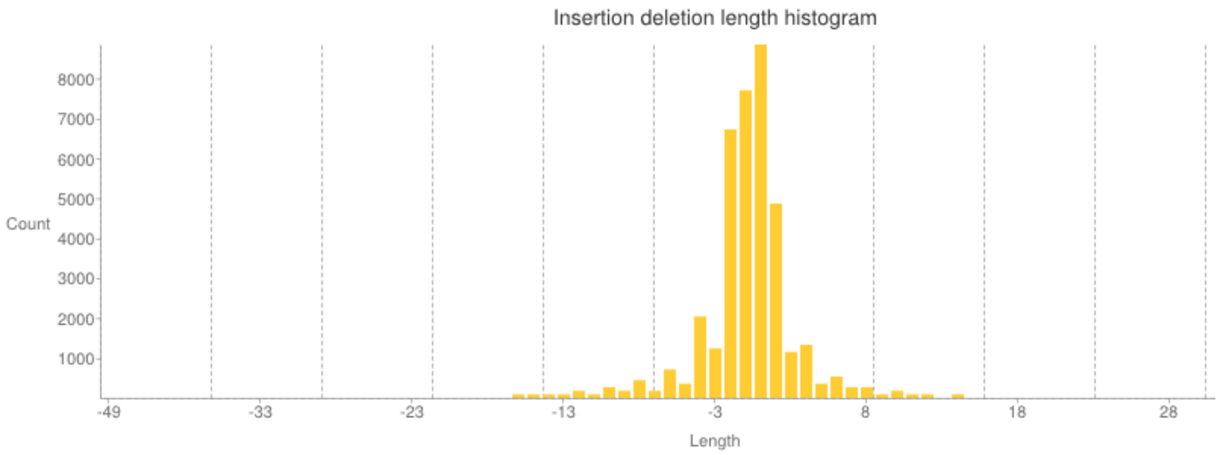

Base changes (SNPs)

	A	C	G	T
A	0	16,262	54,821	19,630
C	18,525	0	11,201	58,946
G	59,129	11,294	0	18,343
T	19,340	54,826	16,331	0

Ts/Tv (transitions / transversions)

Note: Only SNPs are used for this statistic.
Note: This Ts/Tv ratio is a 'raw' ratio. Some people prefer to use a ratio of rates, not observed events. In that case, you need to multiply by 2.0 (since there are twice as many possible transitions than transversions, E[Ts/Tv] ratio is twice the ratio of events).

Transitions	227,722
Transversions	130,926
Ts/Tv ratio	1.7393

All variants:

```
Sample        : pp      Total
Transitions   : 227723  227723
Transversions : 130402  130402
Ts/Tv         : 1.746   1.746
```

Only known variants (i.e. the ones having a non-empty ID field):

```
No results available (empty input?)
```

Frequency of alleles

Note: Number of times an allele appears once (singleton), twice (doubletons), etc.

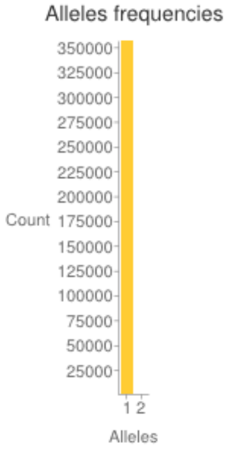

All variants:

Min	1
Max	2
Mean	1.002
Median	1

Standard deviation	0.045
Values	1 2
Count	356681 722

Only known variants (i.e. the ones having a non-empty ID field):

Min	2,147,483,647
Max	-2,147,483,648
Mean	0
Median	0
Standard deviation	0
Values	
Count	

Codon changes

How to read this table:
- Rows are reference codons and columns are changed codons. E.g. Row 'AAA' column 'TAA' indicates how many 'AAA' codons have been replaced by 'TAA' codons.
- Red background colors indicate that more changes happened (heat-map).
- Diagonals are indicated using grey background color
- WARNING: This table may include different translation codon tables (e.g. mamalian DNA and mitochondrial DNA).

	-	AAA	AAC	AAG	AAT	ACA	ACC	ACG	ACT	AGA	AGC	AGG	AGT	ATA	ATC	ATG	ATT	CAA	CAC	CAG	CAT	CCA	CCC	CCG	CCT	CGA	CGC	CGG	CGT	CTA	CTC	CTG	CTT
-		2	5	9	6	7	5	1	2	8	9	7	6	6	7	16	5	4	7	5	6	3	2	1	8	4	1	2	2	6	5	3	5
AAA	8		32	162	49	47				70			1	14				59															
AAC	11	43	3	42	176		21				62				10				12														
AAG	16	171	54	1	52			24	1			79				36				33													
AAT	9	53	160	63					37		1		105				32				38												
ACA	4	43				3	40	108	42	28				92			1					22											
ACC	4		27			44	1	29	132		27				48								14										
ACG	2	1		23		104	28		35			12				82								4									
ACT	4	1			31	55	119	31	1				32				87								20								
AGA	4	95			1	26				1	15	89	18	29												38							
AGC	6		68		1	1	20			22		13	96		21												12						
AGG	8			102			12			80	20	2	26			40												25					
AGT	10				90				55	28	103	15	2			2	25												18				
ATA	6	27				56				34		1			39	65	36													13			
ATC	2		18				39				20			41		26	113														17		
ATG	3			26				89	1	1		23		111	35		37															30	
ATT	6				23				71				24	44	103	21	1																24
CAA	16	69																3	30	136	30	29				85				26			
CAC	5		17															23		21	95		8				50				11		
CAG	20			48														141	27	2	42			7	1			65				11	
CAT	3				37													22	98	25					24				103				22
CCA	10					25												38				1	40	133	53	16				84			
CCC	1						22												18	1		55	1	22	123		9				35		
CCG	1							4												15		153	27	1	39			6				58	
CCT	9								32									1			31	52	109	33	2				14				64
CGA	1									30								93				10					8	32	9	3			
CGC	2										22								58		1		7			13		9	41		18		
CGG												31								81				13		41	11		14			10	
CGT	2												26								100				17	16	44	15				1	15
CTA	3													22				19				56				14				1	33	111	28
CTC	2														26				11				38				10			36		41	147
CTG	2															29				17				49				11		130	43	2	47
CTT	5																52			1	27				54				27	44	139	40	1
GAA	14	183																75															
GAC	5		83																24														
GAG	16			169																47													
GAT	13				156																34												
GCA	6					118																16											
GCC	5						75																12										
GCG	2							35																5									
GCT	6								123																18								
GGA	12									110																17							
GGC	5										62																10						
GGG	4											76																13					
GGT	9												103																20				
GTA	1													79																19			
GTC	2														83																22		
GTG	8															98																29	
GTT	5																149																29
TAA																		5															
TAC	3		11																21														
TAG																				4													
TAT	4				29																54												
TCA	6					16																76											
TCC	3						14																28										
TCG								9																31									
TCT	8								22																54								
TGA	1									1																6							
TGC	5										11																39						
TGG	2											23																36					
TGT	1												18																45				
TTA	4													22																57			
TTC	2														10																43		
TTG	5															46																150	
TTT	3																37																56

	GAA	GAC	GAG	GAT	GCA	GCC	GCG	GCT	GGA	GGC	GGG	GGT	GTA	GTC	GTG	GTT	TAA	TAC	TAG	TAT	TCA	TCC	TCG	TCT	TGA	TGC	TGG	TGT	TTA	TTC	TTG	TTT
-	5	2	4	7	11	2	1	6	5	4		3	4		7	5	1	2	3	3	3	10	3	7	4	7	4	8	5	5	6	4
AAA	155																11															
AAC		68																19														
AAG			119																9													
AAT				116																32												
ACA					87																24											
ACC						51																23										
ACG							29																10									
ACT								85																18								
AGA									79																13							
AGC										35																25						
AGG											62																19					
AGT												83																22				
ATA													85																23			
ATC														75																14		
ATG															120																33	
ATT																108																31
CAA	69																59															
CAC		18																33														
CAG			37																62													
CAT				40																66												
CCA					29																66											
CCC						8																38										
CCG							4																23									
CCT								21																79								
CGA									8																46							
CGC										6																49						
CGG											11																51					
CGT												16																64				
CTA													26																66			
CTC														22																48		
CTG															27																119	
CTT																31																86
GAA	2	51	143	57	34				99				29				28															
GAC	45		38	206		12				63				19				35														
GAG	146	52		57			20		1	1	63				35				35													
GAT	42	176	49	2	1			28				77				23				69												
GCA	48			1	2	51	140	62	21			2	108		1						59											
GCC		19			47	1	41	159		26				50								32										
GCG			26		147	44		40			16				89								17									
GCT			1	48	63	159	43	1				34				101								55								
GGA	91	1		1	24				2	39	89	29	12		1	1									25							
GGC		53				22	1		47		36	117		19												19						
GGG			76				11		107	49	1	50			30												25					
GGT			1	78				33	36	96	33					32												39				
GTA	19				62				11				2	29	104	46													21			
GTC		12				42	1			10			27		41	129														36		
GTG			29				71				14		104	29		56															36	
GTT				26			1	89				26	40	115	48																	39
TAA	2																	2	2	5	1				3				2			
TAC		20															4		6	134	1	5				37				19		
TAG			3														7										4				1	
TAT				37													6	137	8	3				36				59				35
TCA					39												18					56	155	62	16		1		76			
TCC						26												19			41		31	139		21				58		
TCG							8												10		179	33		49			9				86	
TCT								34												52	47	138	38	3				27			1	72
TGA									2								5				3						2		1			
TGC										11								47			1	9			5		16	82		18		
TGG											21								63		1		14		36	9	2	17			33	
TGT												28								67				23	8	73	13	1			1	24
TTA													12				9				58				8					22	136	18
TTC														17				18				46				16			24		25	169
TTG															28				8				68				21		140	30		67
TTT																39				29				74				25	18	165	31	

Amino acid changes

How to read this table:
- Rows are reference amino acids and columns are changed amino acids. E.g. Row 'A' column 'E' indicates how many 'A' amino acids have been replaced by 'E' amino acids.
- Red background colors indicate that more changes happened (heat-map).
- Diagonals are indicated using grey background color
- WARNING: This table may include different translation codon tables (e.g. mamalian DNA and mitochondrial DNA).

	*	-	?	A	C	D	E	F	G	H	I	K	L	M	N	P	Q	R	S	T	V	W	Y
*	17	1					5		2				4				9	7	4			6	7
-	8	448	399	20	15	9	9	9	12	13	18	11	30	16	11	14	9	24	38	15	16	4	5
?																							
A		19		1,000		68	75		99							51			163	351	349		
C	13	6			156			42	39				1					84	62			29	114

D		18		41		384	174		140	58					239						42		104
E	63	30		54		217	291		164			352					122				64		
F		5			41			334			47		197						120		56		47
G	25	30		91	58	133	168		731									246	165		95	25	
H		8				58				193			33		54	32	91	153					99
I		14						45			377	27	77	112	41			35	44	166	268		
K	20	24					274				14	334		36	187		92	149	1	72			
L	25	21						271		38	122		1,511	75		197	37	62	126		146	21	
M		3									183	26	63					24		90	120		
N		20				184				50	42	201			339				168	58			51
P		21		62						49			241			844	55	45	206	83			
Q	121	36					106			129		117	37			37	282	150					
R	59	17			113				182	159	29	197	47	40	1	47	174	549	127	38		70	
S	44	33		107	95			130	118		46		163	2	159	189		108	1,172	137		10	71
T		14		252							228	68		82	58	60		40	134	772			
V		16		266		38	48	75	61		311		156	98							770		
W	99	2			26				21				33					59	15			2	
Y	24	7			96	57		54		75					40				42				274

Changes by chromosome

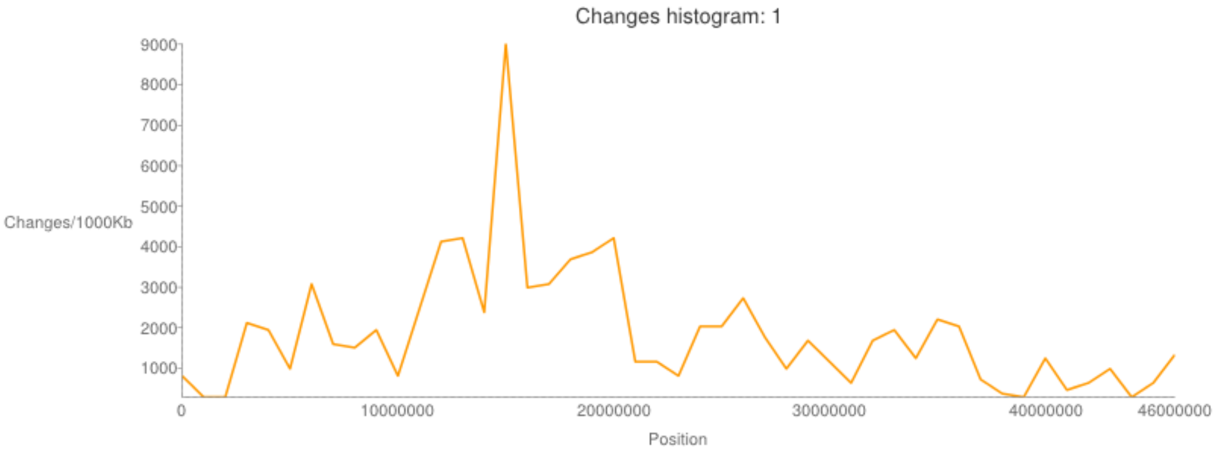


```
Chromosome: 1
```

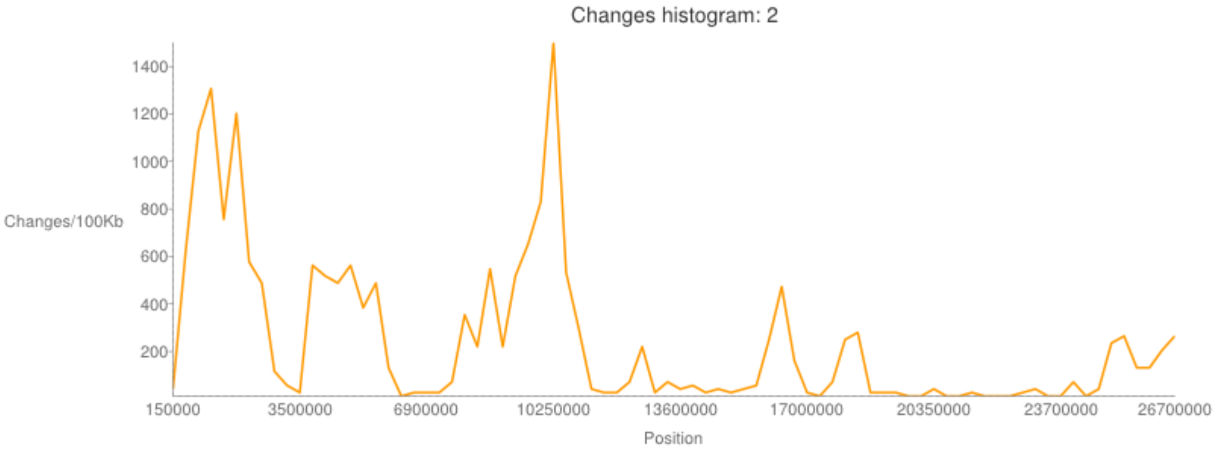


```
Chromosome: 2
```

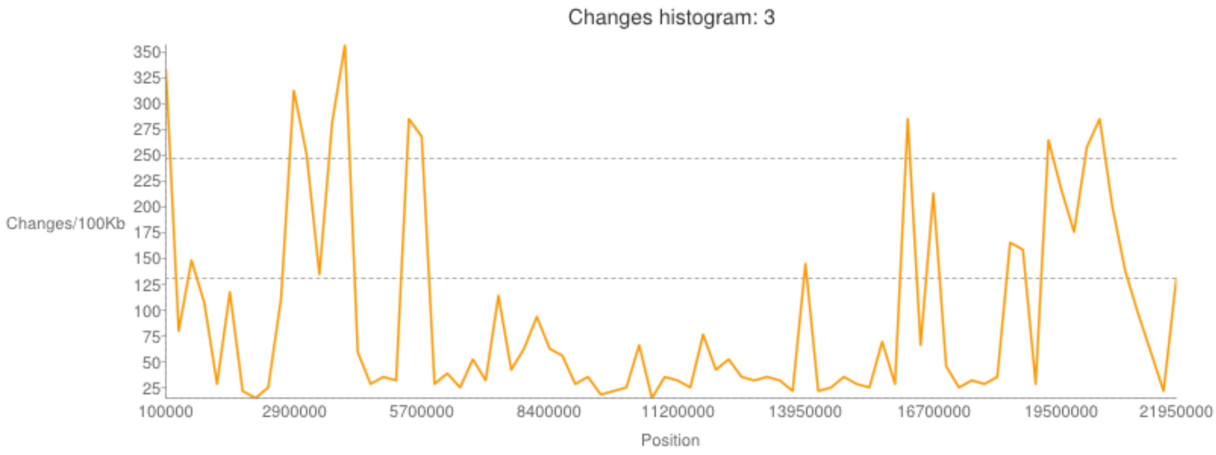

Chromosome: 3

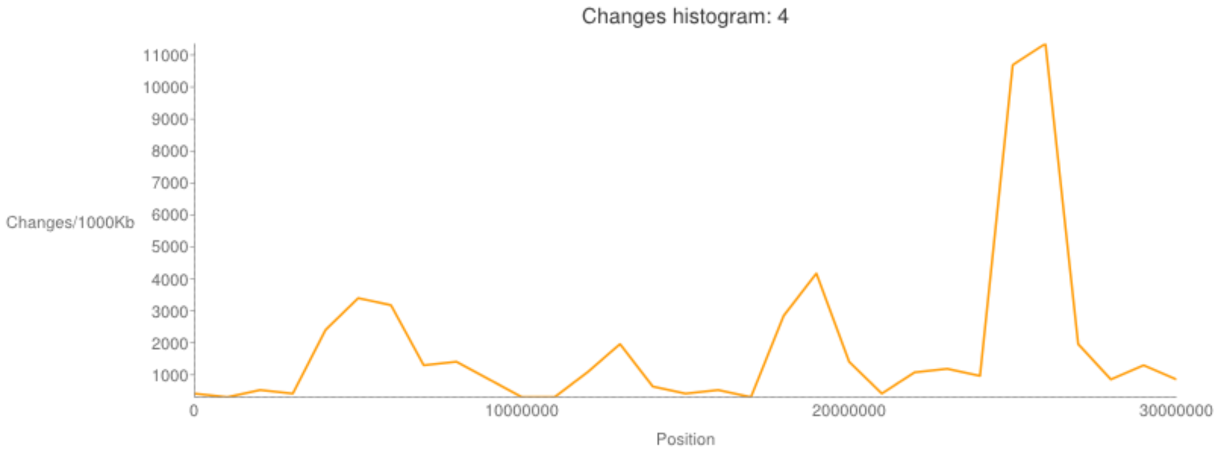

Chromosome: 4

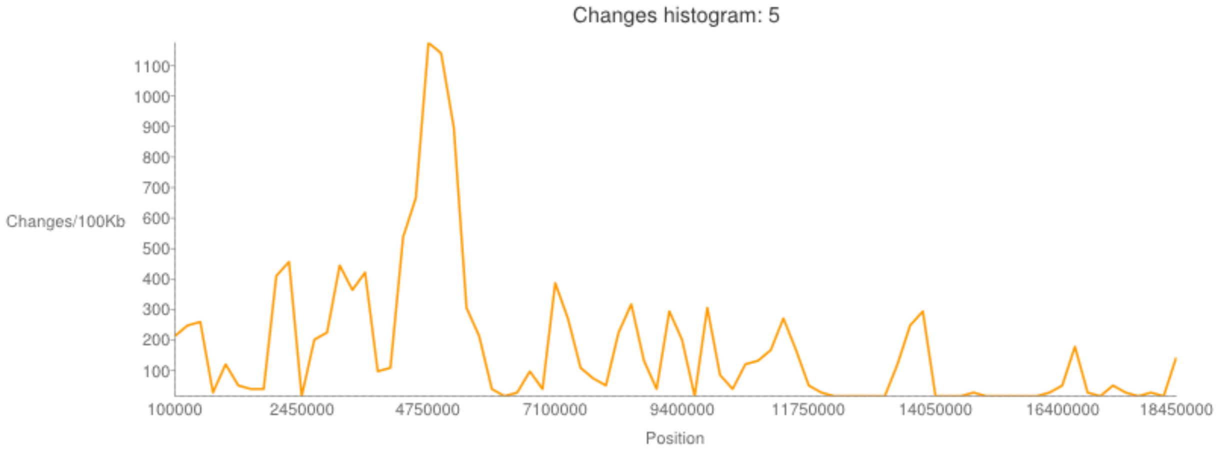

Chromosome: 5

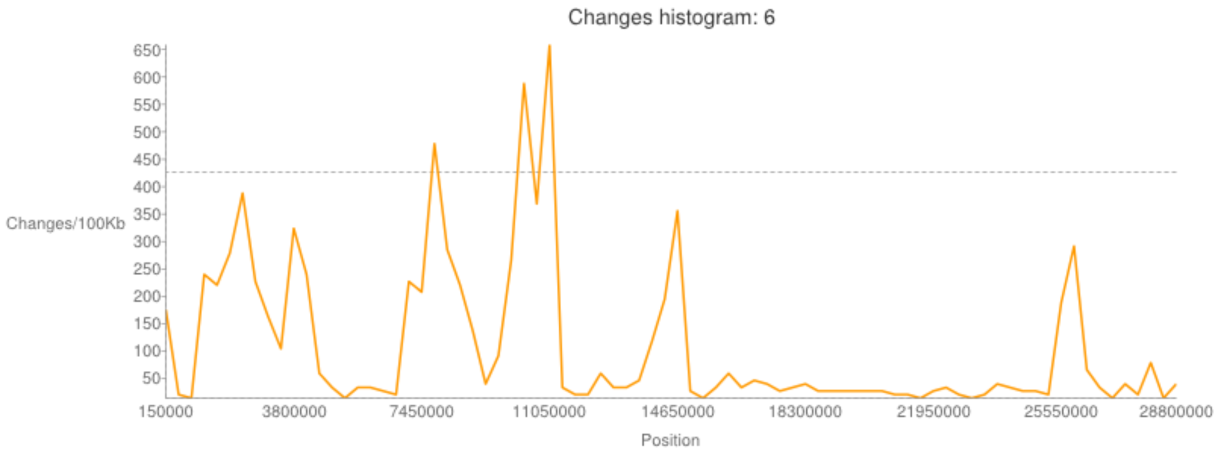

Chromosome: 6

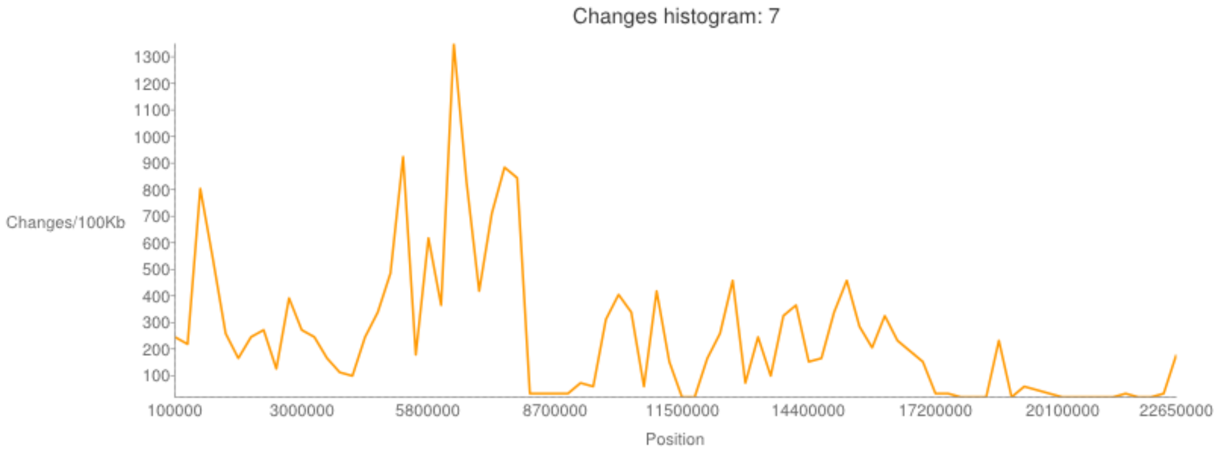

Chromosome: 7

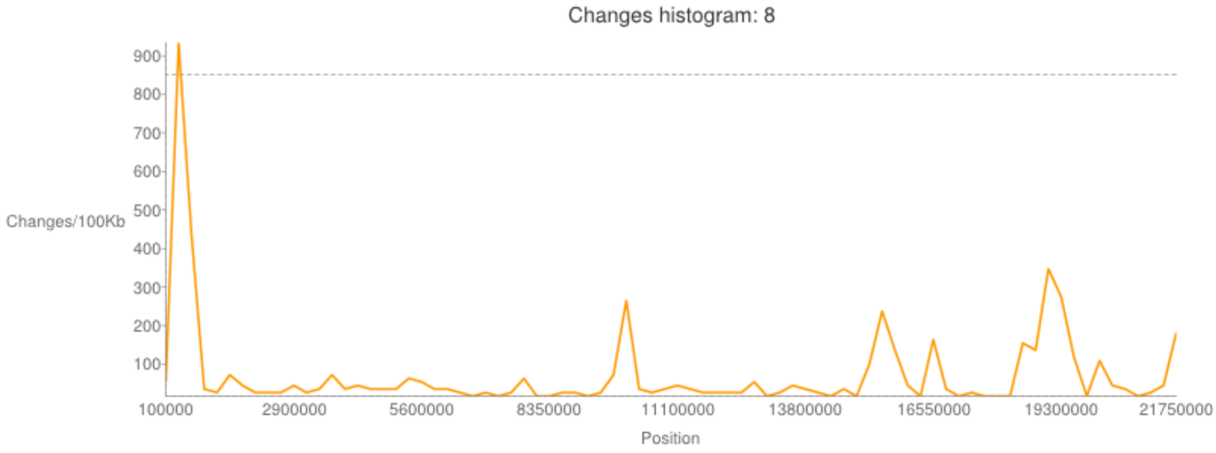

Chromosome: 8

Details by gene

Here you can find a tab-separated table.
