# Supplementary material for: Heterogeneity in the entire genome for three genotypes of peach [Prunus persica (L.) Batsch] as distinguished from sequence analysis of genomic variants
Source: BMC Genomics. 2013 Nov 1;14(1):750. doi: 10.1186/1471-2164-14-750 (PMC4046826; doi:10.1186/1471-2164-14-750)
Supplement: Supplementary file 2 — Additional file 2: Summary file of SnpEff output for 'F8,1-42’. SnpEff_F8.pdf: Summary of statistics of the output of SnpEff 3.0c for the variants present in the peach genotype 'F8,1-42’ in portable document format (PDF). (PDF 806 KB) [file 12864_2013_5471_MOESM2_ESM.pdf]

SnpEff: Variant analysis

Contents

Summary
Change rate by chromosome
Variants by type
Number of variants by impact
Number of variants by functional class
Number of variants by effect
Quality histogram
Coverage histogram
Base change table
Transition vs transversions (ts/tv)
Frequency of alleles
Codon change table
Amino acid change table
Chromosome change plots
Details by gene

Summary

Genome	ppersica139
Date	2012-09-03 21:32
SnpEff version	`SnpEff 3.0c (build 2012-07-30), by Pablo Cingolani`
Command line arguments	`SnpEff  ppersica139 -i vcf /Volumes/USB DISK/VCFs/F8.vcf -s /Volumes/USB DISK/VCFs/F8.html -v`
Warnings	0
Number of lines (input file)	589,305
Number of variants (before filter)	593,720
Filter	
Number of variants filtered out	0
Number of not variants (i.e. reference equals alternative)	0
Number of variants processed (i.e. after filter and non-variants)	593,720
Number of known variants (i.e. non-empty ID)	0 (0%)
Number of effects	971,942
Genome total length	227,252,106
Genome effective length	227,109,400
Change rate	1 change every 382 bases

Change rate details

Chromosome	Length	Changes	Change rate
1	46,877,626	65,517	715
2	26,807,724	218,185	122
3	22,025,550	24,935	883
4	30,528,727	86,579	352
5	18,502,877	16,653	1,111
6	28,902,582	41,228	701
7	22,790,193	35,098	649
8	21,829,753	80,107	272
Total	227,109,400	593,720	382

Number changes by type

Type	Total	Homo	Hetero
SNP	546,542	3,698	542,844
MNP	0	0	0
INS	22,543	8,617	13,926
DEL	24,635	8,476	16,159
MIXED	0	0	0
Interval	0	0	0
Total	593,720	20,791	572,929

Number of effects by impact

Type (alphabetical order)	Count	Percent
HIGH	2,729	0.281%
LOW	20,399	2.099%
MODERATE	29,123	2.996%
MODIFIER	919,691	94.624%

Number of effects by functional class

Type (alphabetical order)	Count	Percent
MISSENSE	28,815	57.82%
NONSENSE	937	1.88%
SILENT	20,084	40.3%

Missense / Silent ratio: 1.4347

Number of effects by type and region

Type

Type (alphabetical order)	Count	Percent
CODON_CHANGE_PLUS_CODON_DELETION	95	0.01%
CODON_CHANGE_PLUS_CODON_INSERTION	131	0.013%
CODON_DELETION	135	0.014%
CODON_INSERTION	63	0.006%
DOWNSTREAM	332,654	34.226%
FRAME_SHIFT	1,284	0.132%
INTERGENIC	147,753	15.202%
INTRON	82,602	8.499%
NON_SYNONYMOUS_CODING	28,699	2.953%
NON_SYNONYMOUS_START	4	0%
SPLICE_SITE_ACCEPTOR	183	0.019%
SPLICE_SITE_DONOR	203	0.021%
START_GAINED	311	0.032%
START_LOST	42	0.004%
STOP_GAINED	947	0.097%
STOP_LOST	70	0.007%
SYNONYMOUS_CODING	20,046	2.062%
SYNONYMOUS_STOP	38	0.004%
UPSTREAM	349,884	35.998%
UTR_3_PRIME	4,602	0.473%
UTR_5_PRIME	2,196	0.226%

Region

Type (alphabetical order)	Count	Percent
DOWNSTREAM	332,654	34.226%
EXON	51,554	5.304%
INTERGENIC	147,753	15.202%
INTRON	82,602	8.499%
SPLICE_SITE_ACCEPTOR	183	0.019%
SPLICE_SITE_DONOR	203	0.021%
UPSTREAM	349,884	35.998%
UTR_3_PRIME	4,602	0.473%
UTR_5_PRIME	2,507	0.258%

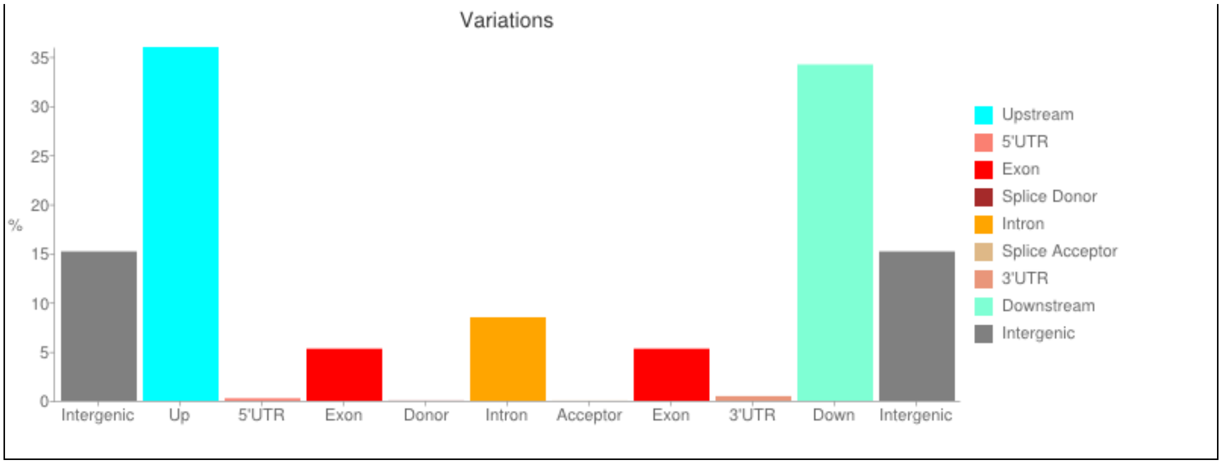

Quality:

**Min**	3
**Max**	225
**Mean**	68.006
**Median**	53
**Standard deviation**	56.375

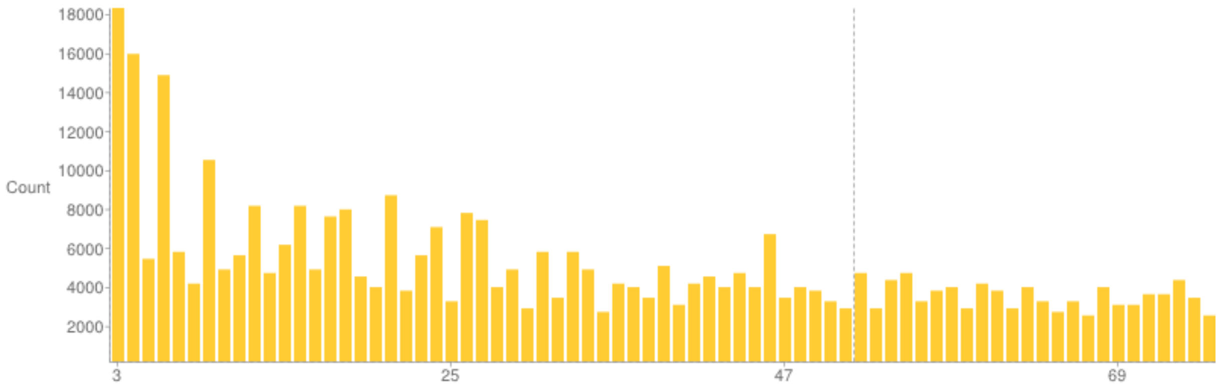

Coverage:

**Min**	1
**Max**	2,100
**Mean**	13.093
**Median**	11
**Standard deviation**	15.681

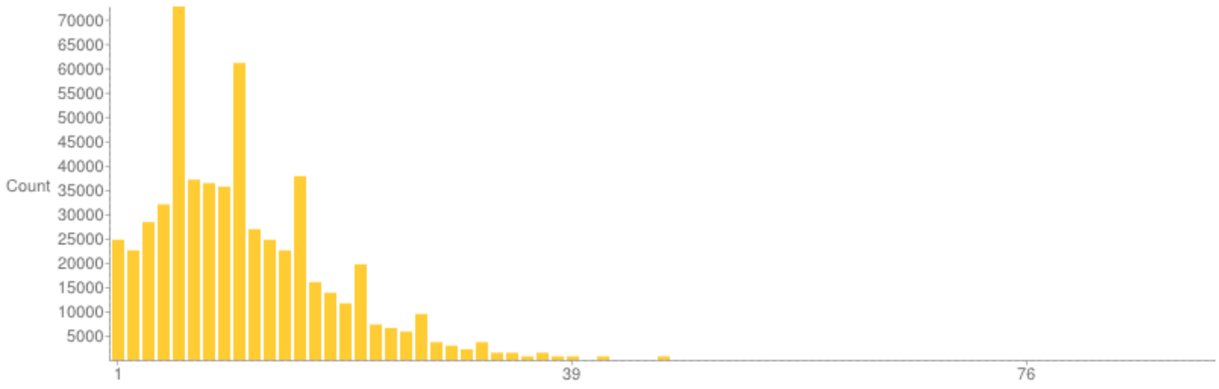

Insertions and deletions length:

**Min**	-45
**Max**	41
**Mean**	-0.43
**Median**	-1
**Standard deviation**	4.983

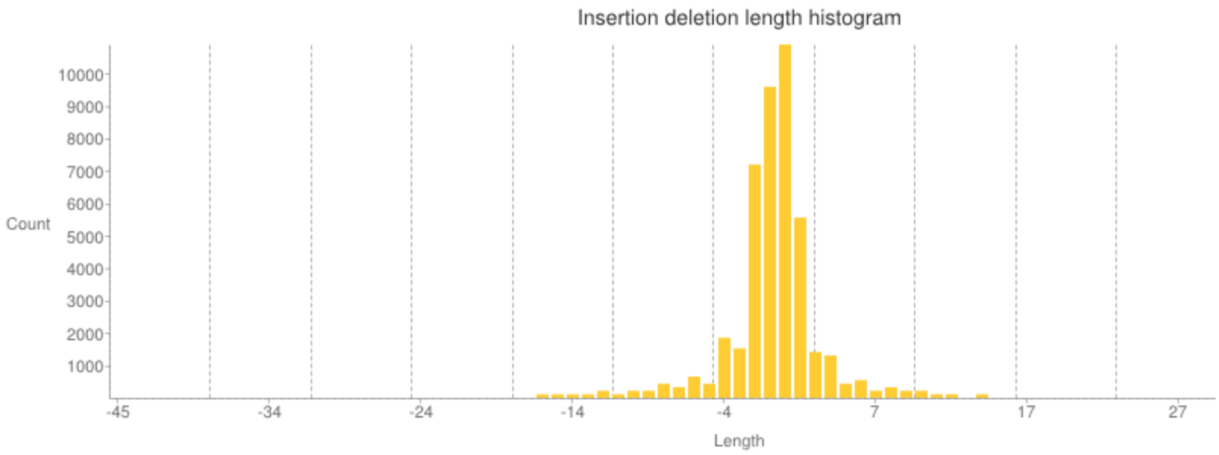

Base changes (SNPs)

	A	C	G	T
A	0	24,674	81,166	30,261
C	30,542	0	17,902	88,797
G	88,082	18,157	0	30,679
T	29,767	81,834	24,681	0

Ts/Tv (transitions / transversions)

Note: Only SNPs are used for this statistic.
Note: This Ts/Tv ratio is a 'raw' ratio. Some people prefer to use a ratio of rates, not observed events. In that case, you need to multiply by 2.0 (since there are twice as many possible transitions than transversions, E[Ts/Tv] ratio is twice the ratio of events).

**Transitions**	339,879
**Transversions**	206,663
**Ts/Tv ratio**	1.6446

All variants:

```
Sample         : pp      Total
Transitions    : 339859  339859
Transversions  : 205804  205804
Ts/Tv          : 1.651   1.651
```

Only known variants (i.e. the ones having a non-empty ID field):

```
No results available (empty input?)
```

Frequency of alleles

Note: Number of times an allele appears once (singleton), twice (doubletons), etc.

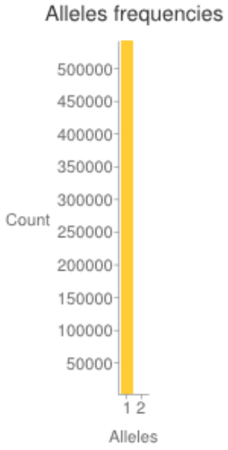

All variants:

**Min**	1
**Max**	2
**Mean**	1.003
**Median**	1

Standard deviation	0.058
Values	1 2
Count	541931 1866

Only known variants (i.e. the ones having a non-empty ID field):

Min	2,147,483,647
Max	-2,147,483,648
Mean	0
Median	0
Standard deviation	0
Values	
Count	

Codon changes

How to read this table:
- Rows are reference codons and columns are changed codons. E.g. Row 'AAA' column 'TAA' indicates how many 'AAA' codons have been replaced by 'TAA' codons.
- Red background colors indicate that more changes happened (heat-map).
- Diagonals are indicated using grey background color
- WARNING: This table may include different translation codon tables (e.g. mamalian DNA and mitochondrial DNA).

	-	AAA	AAC	AAG	AAN	AAT	ACA	ACC	ACG	ACT	AGA	AGC	AGG	AGT	ANA	ANC	ATA	ATC	ATG	ATT	CAA	CAC	CAG	CAT
-		6	2	15		17	5	12	3	4	8	9	15	7	1	1	5	8	10	9	8	6	15	6
AAA	18	1	62	291	1	95	67				151						35				90			
AAC	10	105	2	69		323		45				110						24				24		
AAG	16	321	87	4		137			47	1			194						65				51	
AAN																								
AAT	15	89	279	110		4			1	56		2		178						54				66
ACA	7	86					3	63	256	93	55						190	1						
ACC	7		49				110	1	54	220		51						78						
ACG		1		47			223	50		74			24						148					
ACT	14	1				81	128	223	62	1				57						163				
AGA	4	191					53				1	28	149	38			64							
AGC	12		135					40			55	1	34	205				27						
AGG	8			159		1			23		200	24		60					73					
AGT	7					190		1		78	39	194	28					1		50				
ANA																								
ANC																								
ATA	3	52					119				45						1	62	100	91				
ATC	5		31					64		1		24					93	1	49	235				
ATG	5		1	46					145	1	1		39	1			215	43	2	113				
ATT	5					50	1			125		1		35			94	213	59					
CAA	18	130																			2	52	249	52
CAC	10		49																		48		54	200
CAG	9			78																	240	48	1	85
CAT	8					97															54	175	50	1
CCA	6						53														91			
CCC	5							30														26	1	
CCG	8								18														43	
CCT	7									52											1			55
CGA	1										64										170			
CGC	2											36										85		
CGG	1												66										114	
CGT	6													36										205
CTA	4																53				41			
CTC																		61				24		
CTG	2																		60				25	
CTT	9																			146	1		1	36
GAA	22	318																			133			
GAC	13		178																			44		
GAG	15			309																			93	
GAT	19					268																		75
GCA	6						214																	
GCC	4							123																
GCG	1								50															
GCT	9									216														
GGA	16										172													
GGC	17											130												
GGG	6												164											
GGT	16													186										
GTA	5																172							
GTC	5																	164						
GTG	2																		207					
GTT	8																			319				
NGC																								
TAA																					9			
TAC	4		32																			51		
TAG				1																			10	
TAT	5					59																		116
TCA	10						41																	
TCC	8							38																
TCG	5								13															
TCT	12									56														
TGA																								
TGC	6											34												
TGG	2												35											
TGT	2													36										
TNT																								
TTA	6																44							
TTC	5																	27						
TTG	8																		72					
TTT	8																			49				

	CCA	CCC	CCG	CCT	CGA	CGC	CGG	CGT	CTA	CTC	CTG	CTT	GAA	GAC	GAG	GAT	GCA	GCC	GCG	GCT	GGA	GGC	GGG	GGT
-	3	3	1	9	11	1	1	2	6	3	6	9	14	7	11	7	9	3	2	12	12	8	6	16
AAA													261											
AAC														96										
AAG															224									
AAN																								
AAT																215								
ACA	35																170							
ACC		29																89						
ACG			8																46					
ACT				30																142				
AGA					65																112			
AGC						29																94		
AGG							56																81	
AGT								35																139
ANA																								
ANC																								
ATA									23															
ATC										42														
ATG											52													
ATT												69												
CAA	66			1	138				38				98											
CAC		13				79				20				33										
CAG			32	1			110				34				84									
CAT				43				181				45				76								
CCA	1	77	252	97	39				139								52							
CCC	102		46	240		9				50								25						
CCG	322	47	1	79	1		10				95								11					
CCT	93	208	60	2				28				103								44				
CGA	20					13	71	23	27												18			
CGC		15			35		20	73		27												18		
CGG			20		88	25		39			29												26	
CGT				29	19	78	31	1			1	38												30
CTA	96		1		22					55	200	61												
CTC		42		1		13			84	1	82	296												
CTG	1		68				18		237	89	1	103												
CTT				95				40	84	259	85	1												
GAA													4	91	283	94	71				126			1
GAC													90	1	89	423		24				85		1
GAG													284	97	2	134			42				121	1
GAT													109	361	92	3	1			62				131
GCA	47												89					90	268	142	45			1
GCC		29												38			111	1	62	302		28		
GCG			12												40		292	66		99			26	
GCT				50												92	128	289	108	2			1	64
GGA					47								151	1	2	4	46				1	76	174	75
GGC						23								106			1	50			112	2	78	235
GGG							17						1	1	155	1			28		222	80	1	93
GGT								31								149				66	71	205	74	
GTA									39				36			1	109				26			
GTC										25				19				71	1	1		15		
GTG											42				49	1			131				19	
GTT												61				37				141				33
NGC																								
TAA													4											
TAC														38										
TAG															2									
TAT																68								
TCA	108																59							
TCC		56																49						
TCG			28																16					
TCT				98																53				
TGA					7																1			
TGC						53																16		
TGG							56																45	
TGT								72																29
TNT																								
TTA									105															
TTC										70														
TTG											255													
TTT												98												

	GTA	GTC	GTG	GTT	NGC	TAA	TAC	TAG	TAT	TCA	TCC	TCG	TCT	TGA	TGC	TGG	TGT	TNT	TTA	TTC	TTG	TTT
-	2		5	6	1	1	6	6	2	1	11	1	10	5	7	7	2	1	7	4	6	6
AAA						25																
AAC							31															
AAG								15														
AAN																						
AAT									55													
ACA										53												
ACC											42											
ACG												27										
ACT													62									
AGA														23								
AGC															40							
AGG																33						
AGT																	42					
ANA																						
ANC																						
ATA	150																		40			
ATC		106																		30		
ATG			172																		65	
ATT				228																		70
CAA						93																
CAC							91															
CAG								121														
CAT									156													
CCA										159												
CCC											73											
CCG												36										
CCT													129									
CGA														69								
CGC															86							
CGG																97						
CGT																	131					
CTA	42																		142			
CTC		41																		87		
CTG			55																		245	
CTT				69																		131
GAA	38					66																
GAC		20					58															
GAG			65					85														
GAT		1	1	42					142													
GCA	214									86												
GCC		121									75											
GCG			144									24										
GCT				187									117									
GGA	45		1	1										63								
GGC		36													34							
GGG			56													58						
GGT				80													57					
GTA	1	49	191	83															41			
GTC	65		71	214																49		
GTG	241	69	1	121																	91	
GTT	75	200	104	2																		87
NGC																						
TAA								8	4	4				12					6			
TAC						25	2	12	265		20				54					33		
TAG						10			2			2				3					2	
TAT						18	247	12	1				58				91					72
TCA						47				1	94	306	84	22					153			
TCC							45			103		67	299		33	1				99		
TCG								14		306	63		86			15					127	
TCT									106	121	283	79			1		45				1	115
TGA						8				1					1	7	1		3			
TGC							58				20			11		19	162			35		
TGG								103				26		68	29	1	43				51	
TGT									119				53	7	153	19	2					42
TNT																						
TTA	34					17				83				10					1	46	251	47
TTC		29					39				77		2		26				64	1	48	335
TTG			65					13				115				32			309	75	1	114
TTT				42					62				82				25		30	297	77	

Amino acid changes

How to read this table:
- Rows are reference amino acids and columns are changed amino acids. E.g. Row 'A' column 'E' indicates how many 'A' amino acids have been replaced by 'E' amino acids.
- Red background colors indicate that more changes happened (heat-map).
- Diagonals are indicated using grey background color
- WARNING: This table may include different translation codon tables (e.g. mamalian DNA and mitochondrial DNA).

	*	-	?	A	C	D	E	F	G	H	I	K	L	M	N	P	Q	R	S	T	V	W	Y

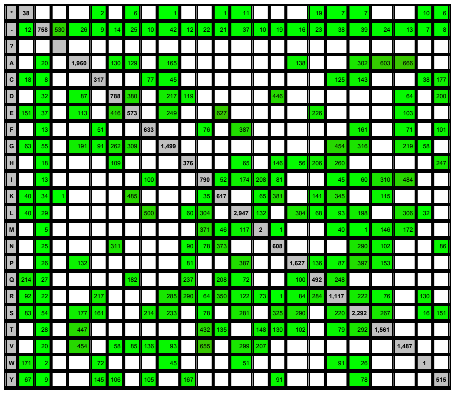

Changes by chromosome

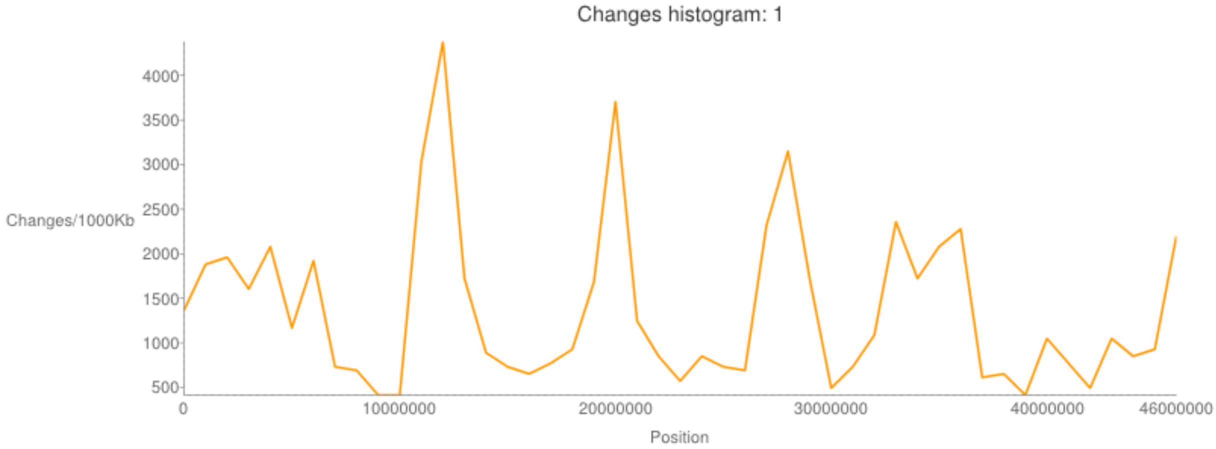

Chromosome: 1

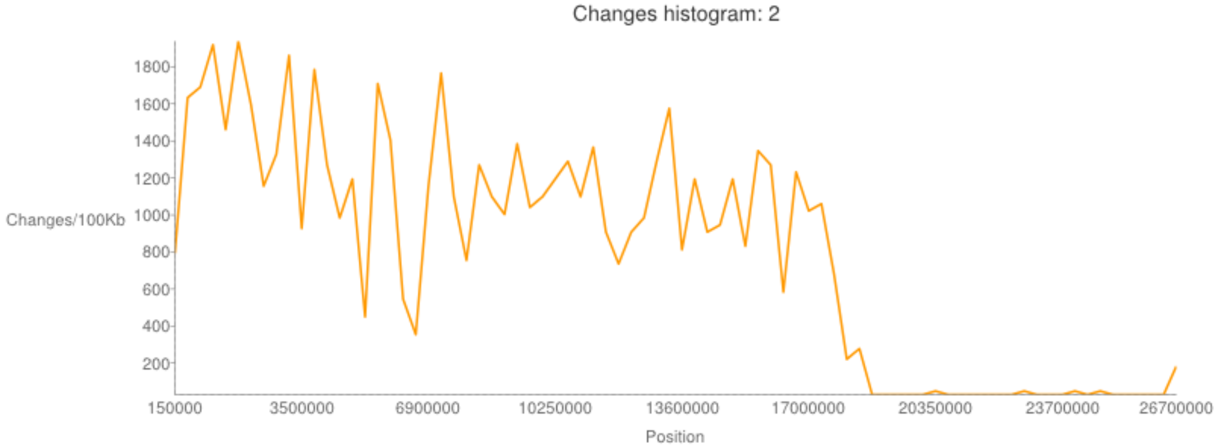

Chromosome: 2

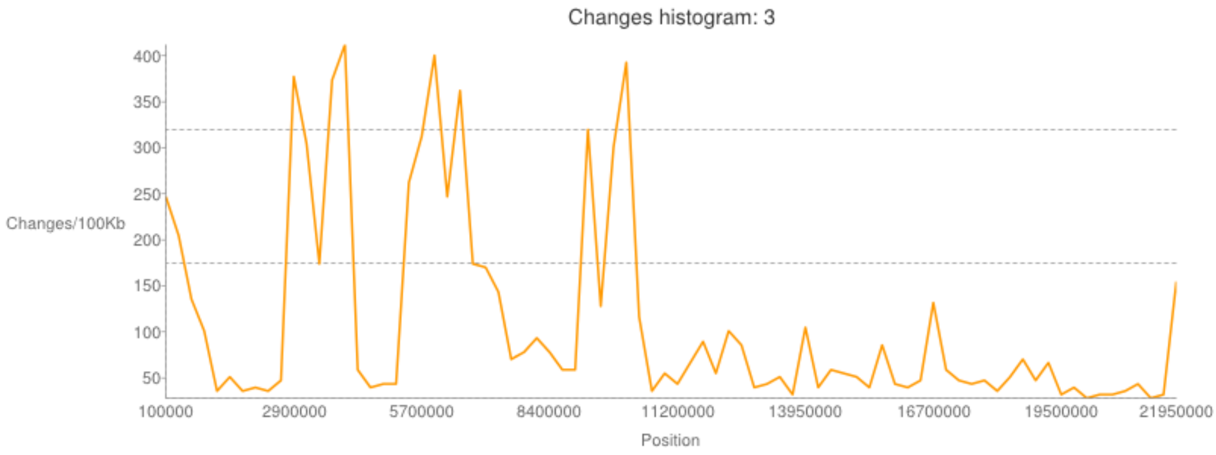

Chromosome: 3

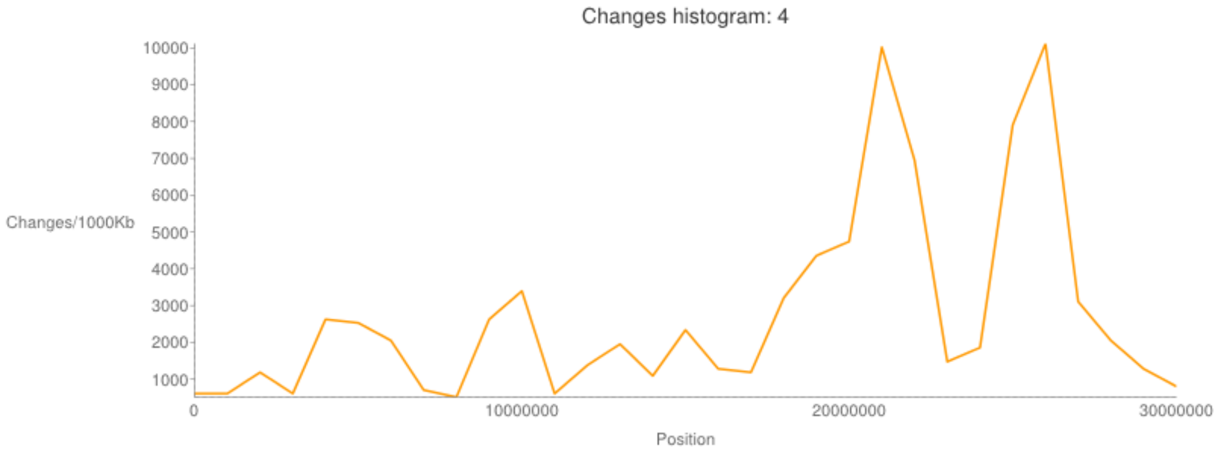

Chromosome: 4

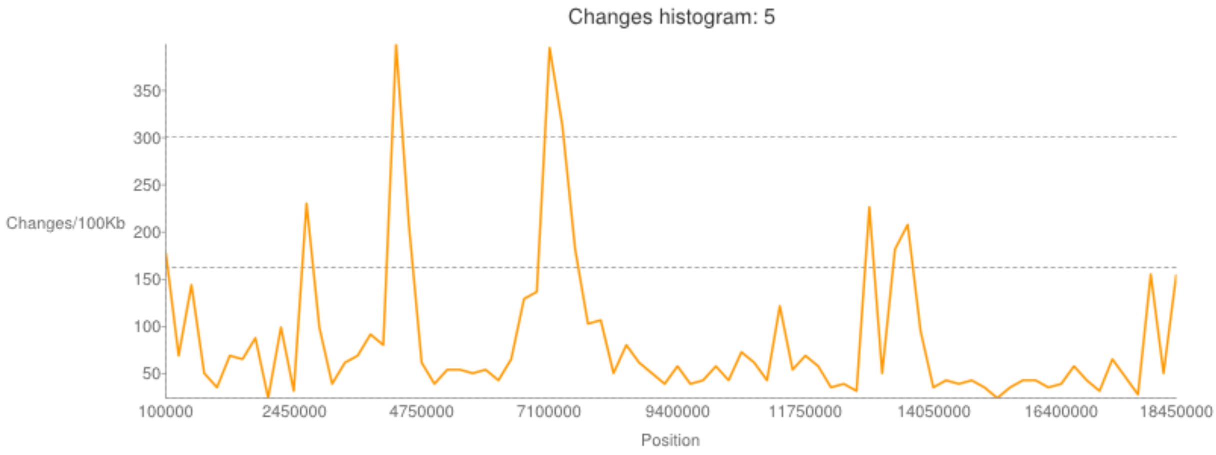

Chromosome: 5

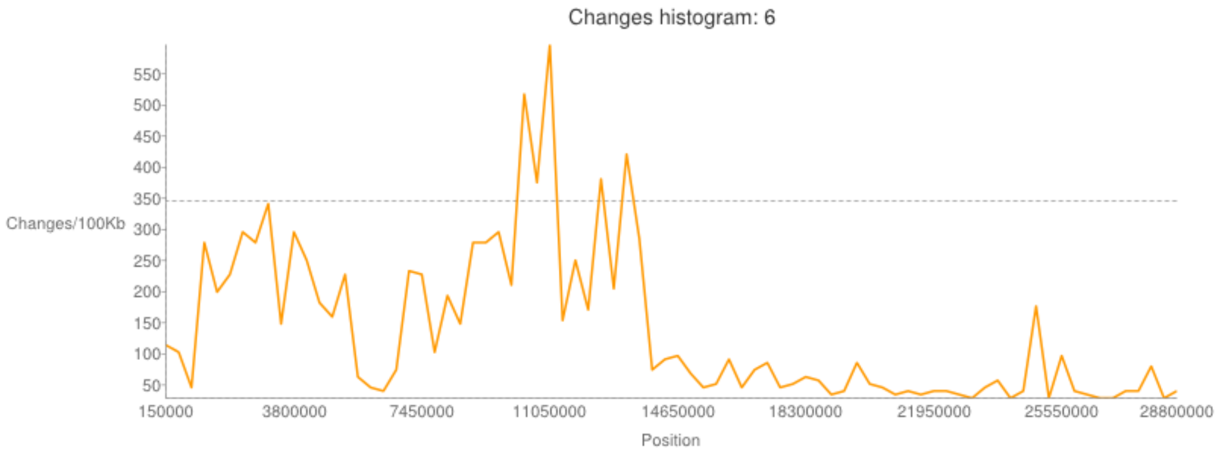

`Chromosome: 6`

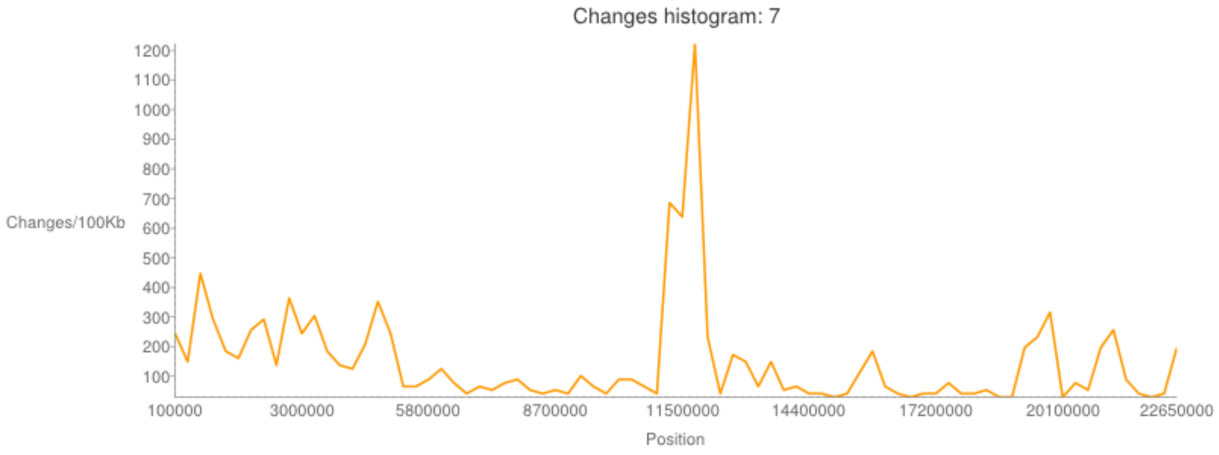

`Chromosome: 7`

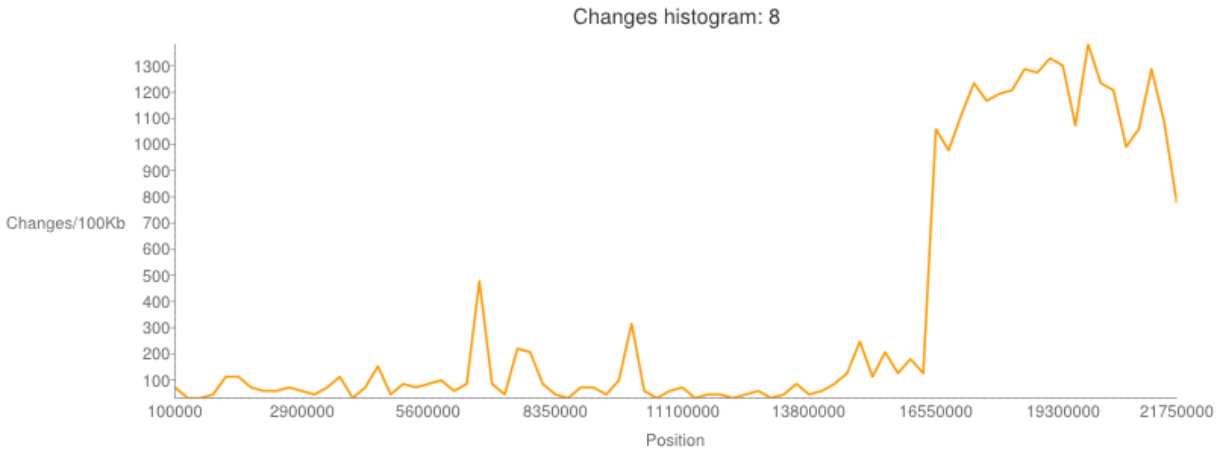

`Chromosome: 8`

Details by gene

Here you can find a tab-separated table.
