# Supplementary material for: Heterogeneity in the entire genome for three genotypes of peach [Prunus persica (L.) Batsch] as distinguished from sequence analysis of genomic variants
Source: BMC Genomics. 2013 Nov 1;14(1):750. doi: 10.1186/1471-2164-14-750 (PMC4046826; doi:10.1186/1471-2164-14-750)
Supplement: Supplementary file 3 — Additional file 3: Summary file of SnpEff output for 'Georgia Belle’. SnpEff_GB.pdf: Summary of statistics of the output of SnpEff 3.0c for the variants present in the peach genotype 'Georgia Belle’ in portable document format (PDF). (PDF 796 KB) [file 12864_2013_5471_MOESM3_ESM.pdf]

SnpEff: Variant analysis

Contents

- Summary
- Change rate by chromosome
- Variants by type
- Number of variants by impact
- Number of variants by functional class
- Number of variants by effect
- Quality histogram
- Coverage histogram
- Base change table
- Transition vs transversions (ts/tv)
- Frequency of alleles
- Codon change table
- Amino acid change table
- Chromosome change plots
- Details by gene

Summary

**Genome**	ppersica139
**Date**	2012-09-03 21:28
**SnpEff version**	`SnpEff 3.0c (build 2012-07-30), by Pablo Cingolani`
**Command line arguments**	`SnpEff  ppersica139 -i vcf /Volumes/USB DISK/VCFs/GB.vcf -s /Volumes/USB DISK/VCFs/GB.html -v`
**Warnings**	0
**Number of lines (input file)**	633,812
**Number of variants (before filter)**	639,062
**Filter**	
**Number of variants filtered out**	0
**Number of not variants (i.e. reference equals alternative)**	0
**Number of variants processed (i.e. after filter and non-variants)**	639,062
**Number of known variants (i.e. non-empty ID)**	0 (0%)
**Number of effects**	1,028,718
**Genome total length**	227,252,106
**Genome effective length**	227,156,785
**Change rate**	1 change every 355 bases

Change rate details

Chromosome	Length	Changes	Change rate
1	46,877,626	101,298	462
2	26,807,724	113,949	235
3	22,025,550	60,176	366
4	30,528,727	92,257	330
5	18,502,877	49,874	370
6	28,902,582	81,503	354
7	22,790,193	56,566	402
8	21,829,753	48,275	452
**Total**	**227,156,785**	**639,062**	**355**

Number changes by type

Type	Total	Homo	Hetero
**SNP**	581,616	2,910	578,706
**MNP**	0	0	0
**INS**	27,515	7,745	19,770
**DEL**	29,931	7,790	22,141
**MIXED**	0	0	0
**Interval**	0	0	0
**Total**	**639,062**	**18,445**	**620,617**

Number of effects by impact

Type (alphabetical order)	Count	Percent
HIGH	2,277	0.221%
LOW	18,032	1.753%
MODERATE	26,029	2.53%
MODIFIER	982,380	95.496%

Number of effects by functional class

Type (alphabetical order)	Count	Percent
MISSENSE	25,729	58.321%
NONSENSE	619	1.403%
SILENT	17,768	40.276%

Missense / Silent ratio: 1.4481

Number of effects by type and region

Type

Type (alphabetical order)	Count	Percent
CODON_CHANGE_PLUS_CODON_DELETION	98	0.01%
CODON_CHANGE_PLUS_CODON_INSERTION	125	0.012%
CODON_DELETION	143	0.014%
CODON_INSERTION	56	0.005%
DOWNSTREAM	351,984	34.216%
FRAME_SHIFT	1,134	0.11%
INTERGENIC	162,860	15.831%
INTRON	79,677	7.745%
NON_SYNONYMOUS_CODING	25,607	2.489%
NON_SYNONYMOUS_START	6	0.001%
SPLICE_SITE_ACCEPTOR	191	0.019%
SPLICE_SITE_DONOR	193	0.019%
START_GAINED	258	0.025%
START_LOST	49	0.005%
STOP_GAINED	635	0.062%
STOP_LOST	75	0.007%
SYNONYMOUS_CODING	17,743	1.725%
SYNONYMOUS_STOP	25	0.002%
UPSTREAM	382,086	37.142%
UTR_3_PRIME	3,863	0.376%
UTR_5_PRIME	1,910	0.186%

Region

Type (alphabetical order)	Count	Percent
DOWNSTREAM	351,984	34.216%
EXON	45,696	4.442%
INTERGENIC	162,860	15.831%
INTRON	79,677	7.745%
SPLICE_SITE_ACCEPTOR	191	0.019%
SPLICE_SITE_DONOR	193	0.019%
UPSTREAM	382,086	37.142%
UTR_3_PRIME	3,863	0.376%
UTR_5_PRIME	2,168	0.211%

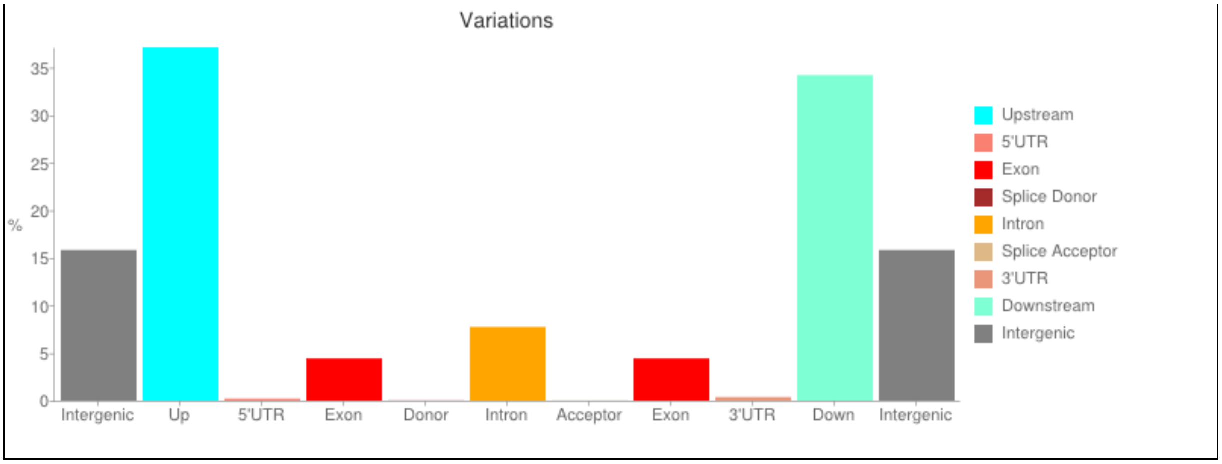

Quality:

Min	3
Max	225
Mean	71.268
Median	61
Standard deviation	53.159

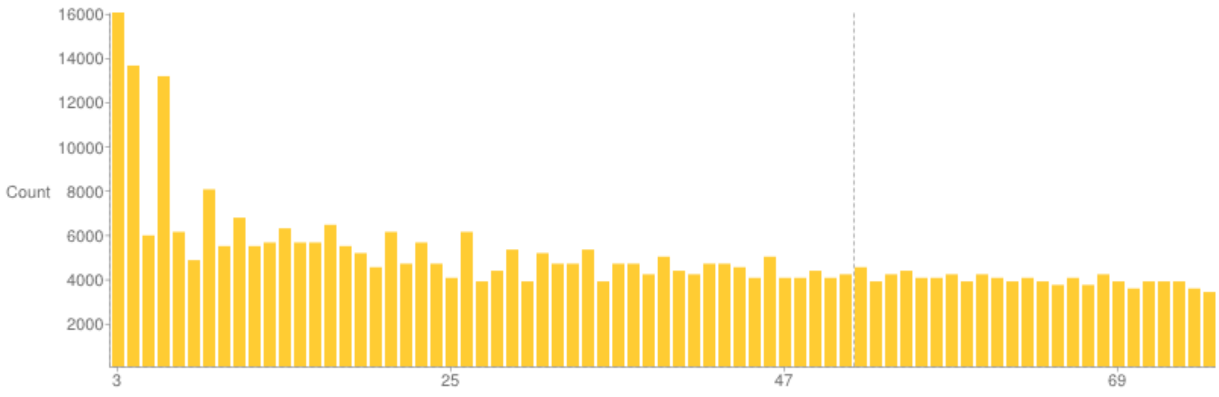

Coverage:

Min	1
Max	1,763
Mean	12.655
Median	11
Standard deviation	11.535

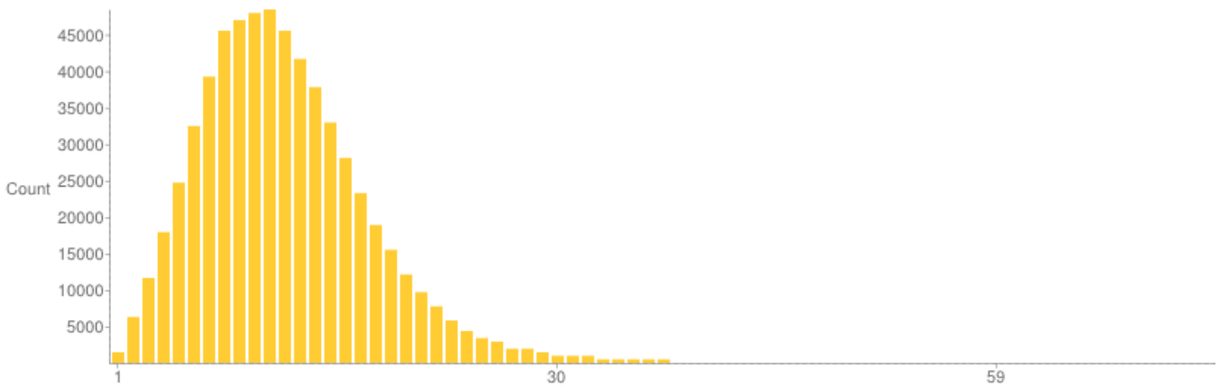

Insertions and deletions length:

Min	-43
Max	27
Mean	-0.459
Median	-1
Standard deviation	5.022

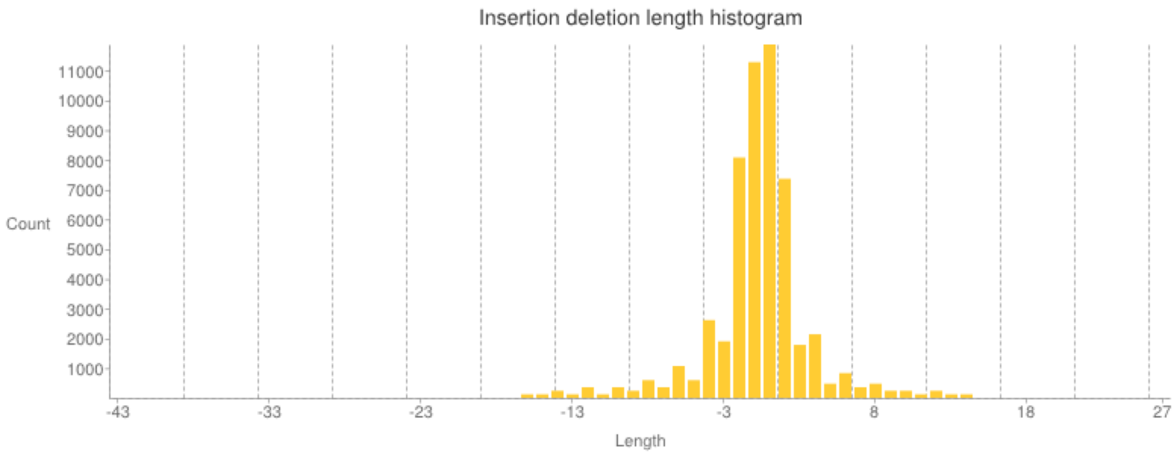

Base changes (SNPs)

	A	C	G	T
A	0	26,510	91,197	29,690
C	29,020	0	18,632	95,952
G	96,058	18,697	0	28,729
T	29,277	91,679	26,175	0

Ts/Tv (transitions / transversions)

Note: Only SNPs are used for this statistic.
Note: This Ts/Tv ratio is a 'raw' ratio. Some people prefer to use a ratio of rates, not observed events. In that case, you need to multiply by 2.0 (since there are twice as many possible transitions than transversions, E[Ts/Tv] ratio is twice the ratio of events).

Transitions	374,886
Transversions	206,730
Ts/Tv ratio	1.8134

All variants:

```
Sample        : /Users/macpro/GenBioPeach/Sequences_DD_F8_GB/BAMs/combined_GB.bam       Total
Transitions   : 374726   374726
Transversions : 205356   205356
Ts/Tv         : 1.825    1.825
```

Only known variants (i.e. the ones having a non-empty ID field):

```
No results available (empty input?)
```

Frequency of alleles

Note: Number of times an allele appears once (singleton), twice (doubletons), etc.

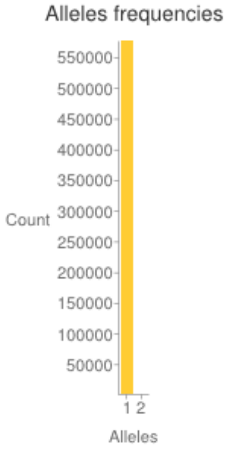

All variants:

Min	1
Max	2
Mean	1.003
Median	1

Standard deviation	0.05
Values	1 2
Count	577160 1461

Only known variants (i.e. the ones having a non-empty ID field):

Min	2,147,483,647
Max	-2,147,483,648
Mean	0
Median	0
Standard deviation	0
Values	
Count	

Codon changes

How to read this table:
- Rows are reference codons and columns are changed codons. E.g. Row 'AAA' column 'TAA' indicates how many 'AAA' codons have been replaced by 'TAA' codons.
- Red background colors indicate that more changes happened (heat-map).
- Diagonals are indicated using grey background color
- WARNING: This table may include different translation codon tables (e.g. mamalian DNA and mitochondrial DNA).

	-	AAA	AAC	AAG	AAT	ACA	ACC	ACG	ACT	AGA	AGC	AGG	AGT	ANC	ATA	ATC	ATG	ATT	CAA	CAC	CAG	CAT	CCA	CCC	CCG	CCT	CGA	CGC	CGG	CGT	CTA	CTC	CTG	CTT
-		4	7	16	16	6	4	4	3	15	5	9	7	1	16	5	26	11	9	5	13	10	4	2	2	9	5		1	5	6	6	4	6
AAA	21	2	74	298	67	64				134			1		33				90															
AAC	12	89	6	69	300		42				103					24	1			24														
AAG	16	306	77	2	104			50	1			137					79	1			59													
AAT	14	69	273	92	2				58		1		188		1			47				55												
ACA	4	63	1			3	84	195	92	57					151			1					27											
ACC	9		39			71		45	208		35					74								25										
ACG	3	1		46		200	56		55			31					159								8									
ACT	4	1			68	102	201	55					53					141								34								
AGA	2	157				41				1	25	144	30		44												59							
AGC	10		128				37			34		34	184			27												23						
AGG	10			152				20		123	20	1	46				64												30					
AGT	8				150				72	28	157	34	1				2	62												33				
ANC																																		
ATA	9	38				112				51		1			1	85	124	74													26			
ATC	5		20		1		54				32				86		35	199														32		
ATG	8		1	47				144	1	3		39			146	48	1	73															47	
ATT	6				53				135				42		82	209	55																	62
CAA	15	110																	3	49	221	50	46				148				32			
CAC	8		36																44		44	180		26				83				19		
CAG	10			76															254	35	1	77			25				111				19	
CAT	6				76														45	172	52	1				47				169		1		45
CCA	6					46													62		1		1	76	241	86	27				109			
CCC	3						24													17			80	3	37	193		19				42		
CCG	2							5													33		254	54		72			11				83	
CCT	10								34													41	87	190	61	2				21			1	103
CGA	1									64									142				12					24	65	18	19			
CGC	2										29									76				14			20	1	14	60		31		
CGG												66									123				12		67	20		27			28	
CGT	7												38									197				29	30	78	22					26
CTA	5														36				37				104				19				1	72	194	49
CTC	6															42				19				45				21			63	2	80	253
CTG	7																48				40				65				16		217	76	2	74
CTT	3																	84			1	39				87				37	78	232	63	1
GAA	20	282																	119															
GAC	13		133																	42														
GAG	17			235																	77													
GAT	23				245																	60												
GCA	9					184																	47											
GCC	3						92																	25										
GCG	2							41																	11									
GCT	7								180																	48								
GGA	15									142																	27							
GGC	8										112																	22						
GGG	3											131																	14					
GGT	7												169																	36				
GTA	6														147																27			
GTC	4															125																25		
GTG	11																168																48	
GTT	6																	251																54
TAA		2																	8															
TAC	5		17																	61														
TAG	1																				8													
TAT	8				52																	110												
TCA	11					26																	95											
TCC	16						24																	35										
TCG	1							15																	34									
TCT	14								47																	98								
TGA	3									1																	9							
TGC	4										17																	67						
TGG	7											30																	78					
TGT	4												38																	91				
TTA	6														39																104			
TTC	2															24																70		
TTG	11																54																249	
TTT	8																	53																118

	GAA	GAC	GAG	GAT	GCA	GCC	GCG	GCT	GGA	GGC	GGG	GGT	GTA	GTC	GTG	GTT	TAA	TAC	TAG	TAT	TCA	TCC	TCG	TCT	TGA	TGC	TGG	TGT	TTA	TTC	TTG	TTT
-	12	3	18	19	14	2	1	11	5	5	6	2	7	3	9	7	4	3	7	12	8	7	4	13	7	3	4	8	4	5	9	6
AAA	274																23															
AAC		118																26														
AAG			236																20													
AAT				217																57												
ACA					149																38											
ACC						75																34										
ACG							38																11									
ACT								144																35								
AGA									132																17							
AGC										76																41						
AGG											104																30					
AGT												152																34				
ANC																																
ATA													147																39			
ATC														108																24		
ATG															174																52	
ATT																224																56
CAA	107																67															
CAC		28																75														
CAG			70																64													
CAT				65																136												
CCA					45																109											
CCC						20																48										
CCG							7																30									
CCT								40																105								
CGA									29																65							
CGC										16																63						
CGG											20																75					
CGT												25																119				
CTA													34																124			
CTC														35																67		
CTG															43																231	
CTT																61																115
GAA	2	91	247	84	62				130				32		2		44															
GAC	97		72	373		15				87				13				41														
GAG	246	90	3	110			42		1	1	109	1			50				42													
GAT	85	323	93	2	1			45				137			2	33				117												
GCA	58			1	1	79	242	107	37			1	151								74											
GCC		23			92		53	237		27				85								51										
GCG			47		267	49		76	1		21				144								17									
GCT			1	60	103	276	82	2				61	1			172								108								
GGA	130		1	3	44				1	61	145	44	28												26							
GGC		81			1	32			83		70	190		17												25						
GGG	2		113				17		170	56	1	84			35												33					
GGT		1	1	143				48	70	187	63					47												57				
GTA	31	1		2	117			1	24				2	52	177	62													34			
GTC		18				66				22			46	1	73	191														39		
GTG			47				127				26		172	51	1	105															61	
GTT				39				143				38	69	200	99																	55
TAA	5																	1	5	3	2				5				2			
TAC		32															6		11	249		20				59				48		
TAG			5														8			1			1				7				2	
TAT				72													10	238	11			1		71				103			1	60
TCA					63												30					64	275	89	8		1		106			
TCC						47												30			81		52	215		20	1			74		
TCG							15												11		314	51	1	82			19				107	
TCT								54												85	92	218	59	2		1		47			2	136
TGA									2								7				3					1	6		2			
TGC										16								55				17			11		20	162		24		
TGG											43							1	66		1	1	24		47	17	1	26			41	
TGT												35								104				43	9	132	26	1				38
TTA													34				12				95				8				1	38	253	40
TTC														31				35				70			1	24			54	1	66	326
TTG															75				15		1		125				40		269	63	2	83
TTT																52				60				101				44	33	269	62	

Amino acid changes

How to read this table:
- Rows are reference amino acids and columns are changed amino acids. E.g. Row 'A' column 'E' indicates how many 'A' amino acids have been replaced by 'E' amino acids.
- Red background colors indicate that more changes happened (heat-map).
- Diagonals are indicated using grey background color
- WARNING: This table may include different translation codon tables (e.g. mamalian DNA and mitochondrial DNA).

	*	-	?	A	C	D	E	F	G	H	I	K	L	M	N	P	Q	R	S	T	V	W	Y
*	25	4			1		10		2			2	6				16	10	6			13	5
-	18	659	476	28	11	22	30	11	18	15	32	20	35	26	23	17	22	35	44	17	26	4	15
?																							
A		21		1,666		84	106		148							131			250	497	553		

C	20	8			295			62	51									158	115			46	159
D		36		61		698	347		224	102					378						48		158
E	86	37		104		375	498		242			517					196				84		
F	1	10			68			596			77		403						171		83		95
G	26	33		142	82	228	247		1,225									372	281		127	33	
H		14				93				353			65		112	73	185	252					211
I		20						80			736	38	159	214	74			52	74	301	479		
K	43	37					510				34	608		79	322		149	271	1	115			
L	35	38						406		58	201		2,690	102		301	78	93	221		282	40	
M		8									267	47	99	1	1			42		145	174		
N		26				335				79	72	319		1	581				292	100			83
P		21		112						58			338			1,437	96	78	292	109			
Q	131	25					177			211		186	51			71	479	259					
R	82	22			182				326	273	44	309	104	64		67	265	934	188	61		105	
S	49	60		179	143			210	228		89		215	2	278	262		186	1,937	221		21	115
T		20		406							367	111		159	108	94		88	206	1,367			
V		27		454		60	78	94	110		523		249	168							1,301		
W	113	7			43				43				41					108	26			1	1
Y	38	13			162	104		108		171			1		69				92				487

Changes by chromosome

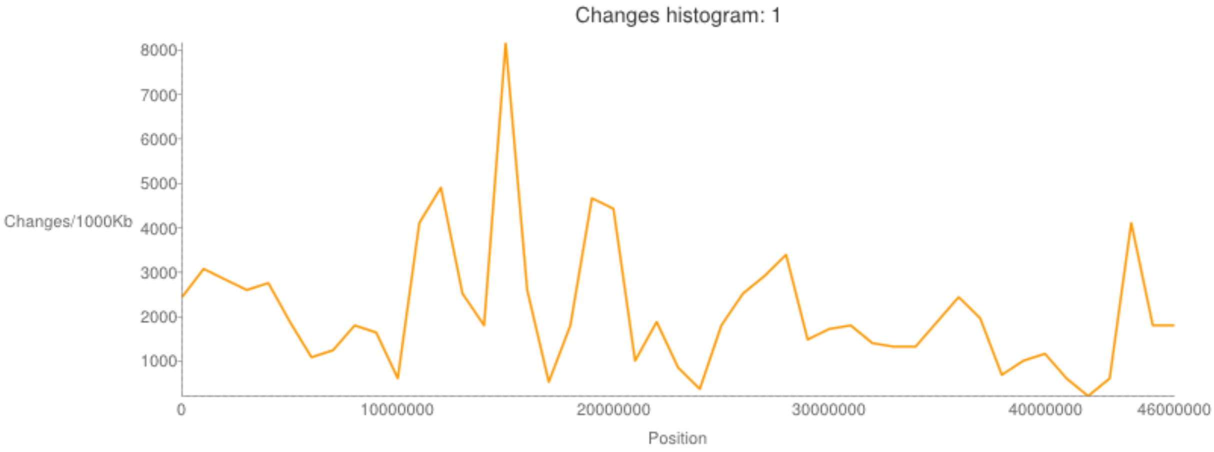

Chromosome: 1

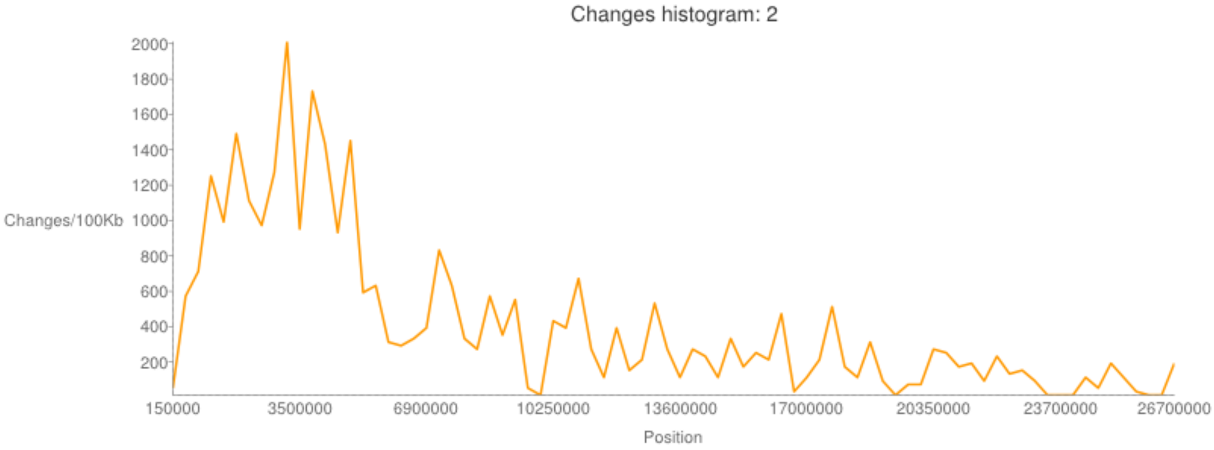

Chromosome: 2

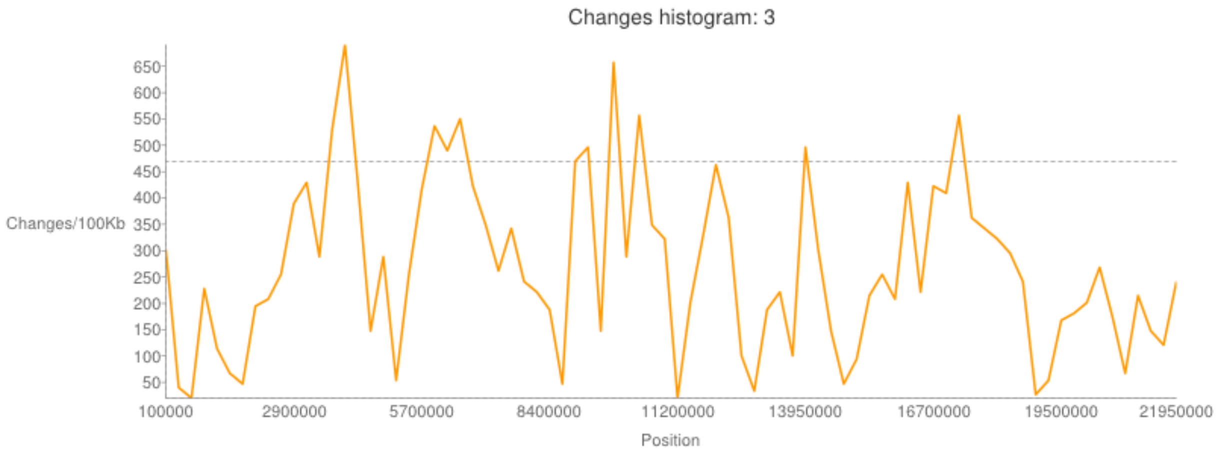

Chromosome: 3

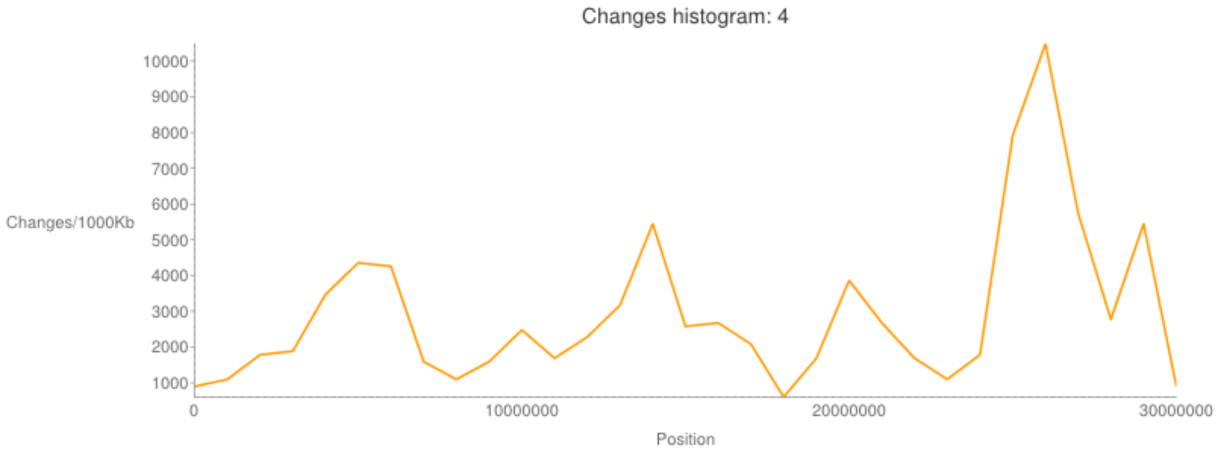

Chromosome: 4

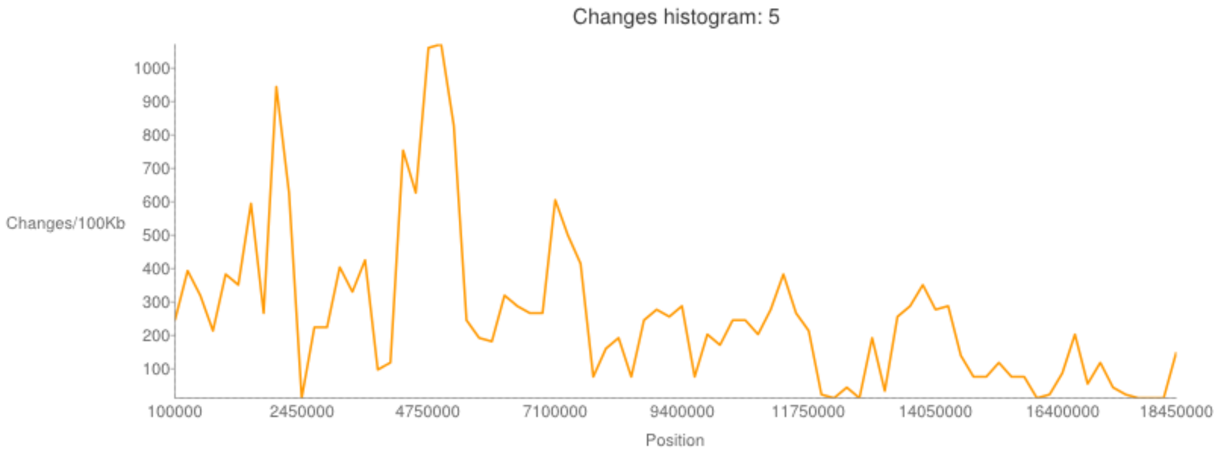

Chromosome: 5

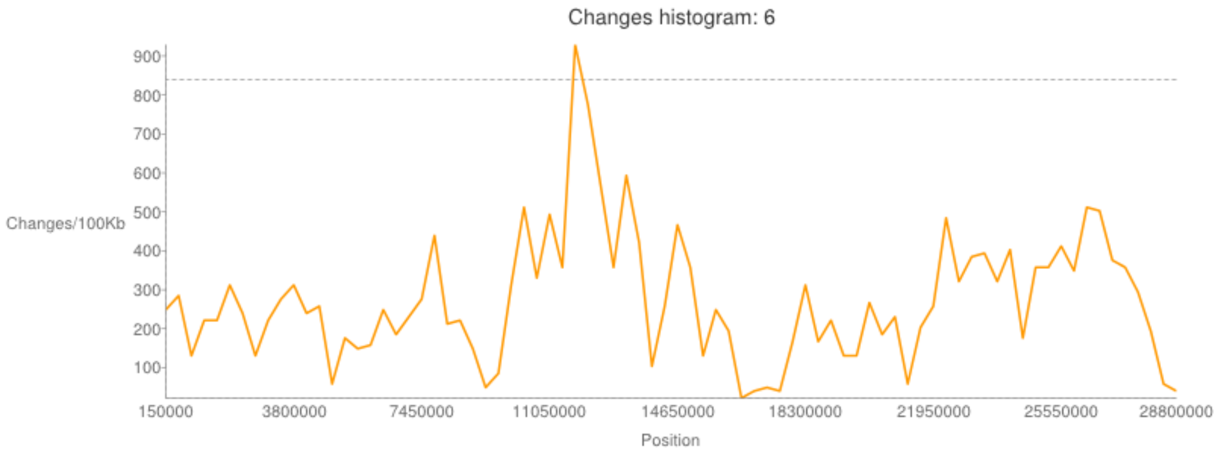

Chromosome: 6

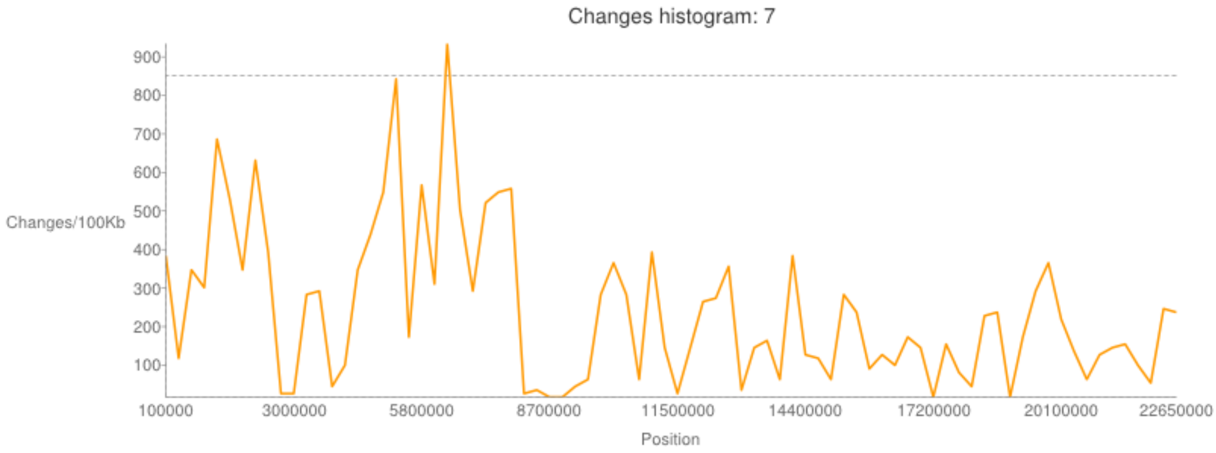

Chromosome: 7

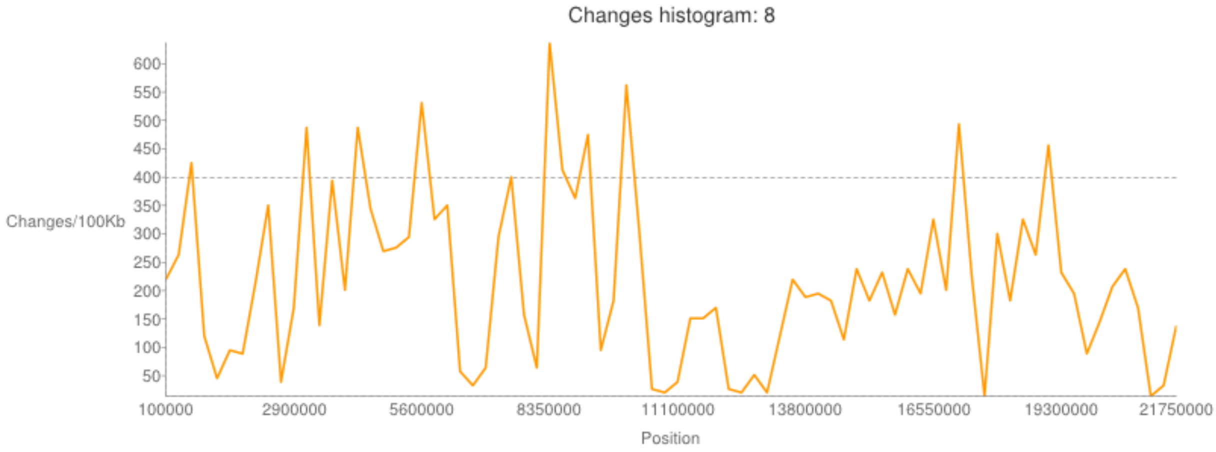

Chromosome: 8

Details by gene

Here you can find a tab-separated table.
